# Electrostatic [FeFe]-hydrogenase–carbon nitride assemblies for efficient solar hydrogen production†

**DOI:** 10.1039/d4sc00640b

**Published:** 2024-03-13

**Authors:** Yongpeng Liu, Carolina Pulignani, Sophie Webb, Samuel J. Cobb, Santiago Rodríguez-Jiménez, Dongseok Kim, Ross D. Milton, Erwin Reisner

**Affiliations:** a Yusuf Hamied Department of Chemistry, University of Cambridge Cambridge CB2 1EW UK reisner@ch.cam.ac.uk; b Department of Inorganic and Analytical Chemistry, University of Geneva Geneva 41211 Switzerland; c National Centre of Competence in Research (NCCR) Catalysis, University of Geneva Geneva 41211 Switzerland

## Abstract

The assembly of semiconductors as light absorbers and enzymes as redox catalysts offers a promising approach for sustainable chemical synthesis driven by light. However, achieving the rational design of such semi-artificial systems requires a comprehensive understanding of the abiotic–biotic interface, which poses significant challenges. In this study, we demonstrate an electrostatic interaction strategy to interface negatively charged cyanamide modified graphitic carbon nitride (^NCN^CN_*X*_) with an [FeFe]-hydrogenase possessing a positive surface charge around the distal FeS cluster responsible for electron uptake into the enzyme. The strong electrostatic attraction enables efficient solar hydrogen (H_2_) production *via* direct interfacial electron transfer (DET), achieving a turnover frequency (TOF) of 18 669 h^−1^ (4 h) and a turnover number (TON) of 198 125 (24 h). Interfacial characterizations, including quartz crystal microbalance (QCM), photoelectrochemical impedance spectroscopy (PEIS), intensity-modulated photovoltage spectroscopy (IMVS), and transient photocurrent spectroscopy (TPC) have been conducted on the semi-artificial carbon nitride-enzyme system to provide a comprehensive understanding for the future development of photocatalytic hybrid assemblies.

## Abstract 2

Solar hydrogen production has been established by interfacing cyanamide modified graphitic carbon nitride (^NCN^CN_*X*_) with [FeFe]-hydrogenase *via* electrostatic interaction. QCM, PEIS, IMVS, and TPC reveal the importance of specific interaction.

## Graphical abstract

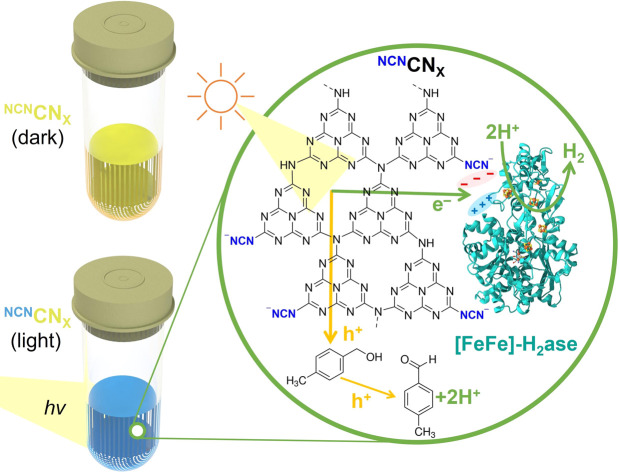

## Introduction

Converting solar energy into clean chemical fuels, such as molecular hydrogen (H_2_), holds promise for advancing the concept of a circular economy.^1^ Among various photocatalysts, carbon nitride (CN_*X*_) has emerged as a particularly attractive candidate due to its unique advantages, including visible light absorption, cost-effective fabrication, scalability, and low toxicity.^2^ To further enhance the photocatalytic performance of CN_*X*_, significant efforts have been devoted to chemical modifications and the incorporation of co-catalysts.^3^ The introduction of ionic cyanamide functional groups into CN_*X*_ (^NCN^CN_*X*_) has demonstrated substantial improvements in charge separation and photocatalytic activity, attributed to the prolonged lifetimes of photogenerated electrons.^4,5^ Moreover, the negatively charged cyanamide group provides a versatile platform for potential electrostatic interactions with co-catalysts.

Nature has evolved enzymes as highly specific biological catalysts to facilitate essential processes in living organisms. Among these enzymes, hydrogenases (H_2_ases) stand out for their remarkable ability to catalyze the interconversion of protons and H_2_ with high efficiency at near-zero overpotential under mild conditions,^6^ surpassing the capabilities of synthetic catalysts.^7^ H_2_ases can be classified into three main types based on their metal cofactors: [NiFe]-H_2_ase, [FeFe]-H_2_ase, and [Fe]-H_2_ase, with [FeFe]-H_2_ase being generally the most active for the hydrogen evolution reaction (HER).^6^ The extensive investigation of H_2_ases as model biocatalysts^8^ has not only inspired the design of artificial systems such as synthetic Fe_2_S_2_(CO)_6_ catalysts that mimic the active site of the Fe_2_S_2_ subunit of the [FeFe]-H_2_ase (Fig. S1†),^9,10^ but also paves the way for developing biohybrid assemblies in semi-artificial photosynthesis systems.^11,12^

By interfacing CN_*X*_ with H_2_ase, we combine the strengths of both artificial and biological approaches, resulting in unique properties that neither system can achieve individually.^11,12^ This integration opens up new avenues for exploring synergistic effects and unlocking unprecedented possibilities in solar energy conversion and catalysis. The activation of [FeFe]-H_2_ase by light can be considered as a model for the development of efficient bio-hybrid systems. Such a photocatalytic system has thus far been demonstrated using either toxic and expensive CdTe nanocrystals^13^ or carbon dots with a low turnover number (TON) of 20 000 over 24 h.^14^ Direct electron transfer (DET) between graphitic carbon nitride (g-C_3_N_4_) and [NiFeSe]-H_2_ase has been established with non-specific interactions, resulting in a turnover frequency (TOF) of 4117 h^−1^ over 4 h.^15^ Subsequent improvements involved the incorporation of a non-diffusional electron mediator, TiO_2_, between g-C_3_N_4_ and [NiFeSe]-H_2_ase, leading to an enhanced TON (4 h) of 80 000.^16^ In addition to its application in H_2_ase systems, CN_*X*_ has predominantly been utilized for the regeneration of NADH in mediated electron transfer (MET) processes involving formate dehydrogenase^17^ or alcohol dehydrogenase.^18^

In this work, we present an approach for biological integration with CN_*X*_ by demonstrating the electrostatic interaction with enzymes to form a functional biohybrid assembly. This method establishes a benchmark for solar H_2_ production, complemented by comprehensive interfacial characterizations utilizing a quartz crystal microbalance (QCM), photoelectrochemical impedance spectroscopy (PEIS), intensity-modulated photovoltage spectroscopy (IMVS), and transient photocurrent spectroscopy (TPC). Specifically, we coupled negatively charged ^NCN^CN_*X*_ with H_2_ases containing different surface charges for *in vitro* photocatalytic H_2_ production without an external electron relay. The adsorption process of H_2_ases on ^NCN^CN_*X*_ is quantified by QCM, whereas PEIS provides insights into the charge carrier dynamics at the biomaterial interface.

## Results and discussion

The ^NCN^CN_*X*_ photocatalysts were synthesized using melamine and potassium thiocyanate following previously published methods.^4,19^ Detailed synthesis procedures and characterizations, including scanning electron microscopy (SEM), attenuated total reflectance Fourier-transform infrared (ATR-FTIR) spectroscopy, fluorescence spectroscopy, and ultraviolet-visible spectroscopy are provided in the ESI (Fig. S2–S5).† [FeFe]-H_2_ase from *Clostridium pasteurianum* (*CpI*, heterologously produced in *Escherichia coli*) and [NiFeSe]-H_2_ase from *Desulfovibrio vulgaris* Hildenborough (*Dv*H) were expressed and purified under anaerobic conditions.^20,21^

**Fig. 1:**
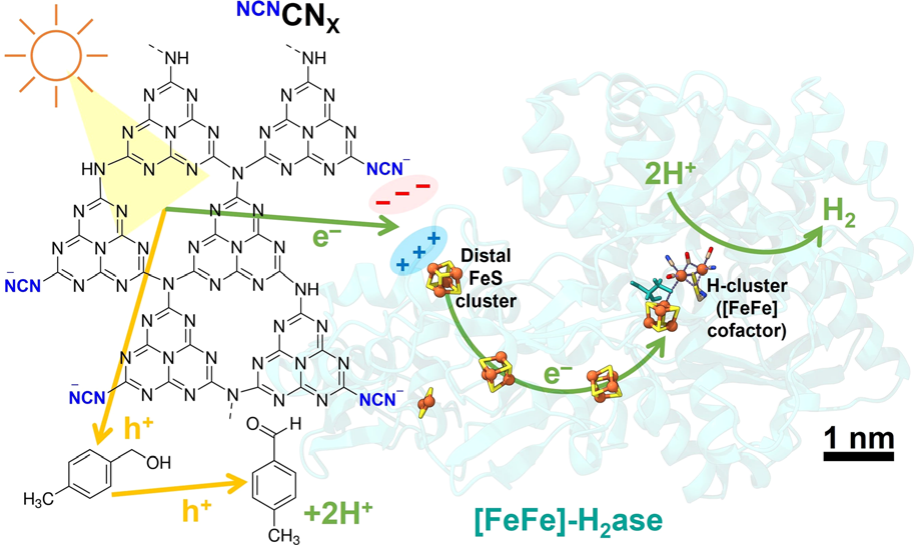
Schematic of photocatalytic H_2_ evolution coupled with alcohol oxidation to aldehyde using an electrostatic ^NCN^CN_X_|[FeFe]-H_2_ase (*CpI*, PDB: 4XDC) assembly. Scale bar refers to enzyme and CN_*X*_ is not shown to scale.

Fig. 1 illustrates the hypothesis that the negatively charged ^NCN^CN_*X*_ possesses the ability to activate enzymes with positively charged electron entry points, such as *CpI* [FeFe]-H_2_ase.^22^ Additionally, the unique property of ^NCN^CN_*X*_ in converting alcohols selectively into aldehydes offers an opportunity to monitor the clean oxidation reaction of 4-methylbenzyl alcohol (4-MBA) to 4-methylbenzaldehyde (*p*-tolualdehyde), allowing the quantification of stoichiometry from the products resulting from oxidation and reduction.^5,23^

To construct a photocatalytic system for solar H_2_ production coupled with selective alcohol oxidation, we interfaced [FeFe]-H_2_ase with ^NCN^CN_*X*_ in the presence of 50 mM 4-MBA in 1 mL aqueous MOPS buffer solution (0.1 M, pH 7). To determine the surface charge of ^NCN^CN_*X*_, zeta potential measurements were performed. Notably, the presence of light-induced blue radicals (absorption band from 500–750 nm, Fig. S5†)^5,24^ did not impact the surface charge of ^NCN^CN_*X*_ (Fig. S6†), indicating that these radicals are long-lived and deeply trapped photoelectrons.^23,25^ The negatively charged surface is primarily attributed to the cyanamide group, which maintains a negative zeta potential even at a pH below 2 (Fig. S7†). *CpI* [FeFe]-H_2_ase was selected due to its distal FeS cluster ([4Fe-4S]) being surrounded by a positively charged region containing surface arginine and lysine residues. *In vivo*, this distal FeS cluster region is thought to interact with the negatively charged region of ferredoxin for electron transfer.^22^ By constructing electrostatic ^NCN^CN_*X*_|[FeFe]-H_2_ase assemblies, we achieve efficient solar H_2_ production, with ^NCN^CN_*X*_ mimicking the role of ferredoxin to deliver electrons directly into [FeFe]-H_2_ase (Fig. 1).

**Fig. 2:**
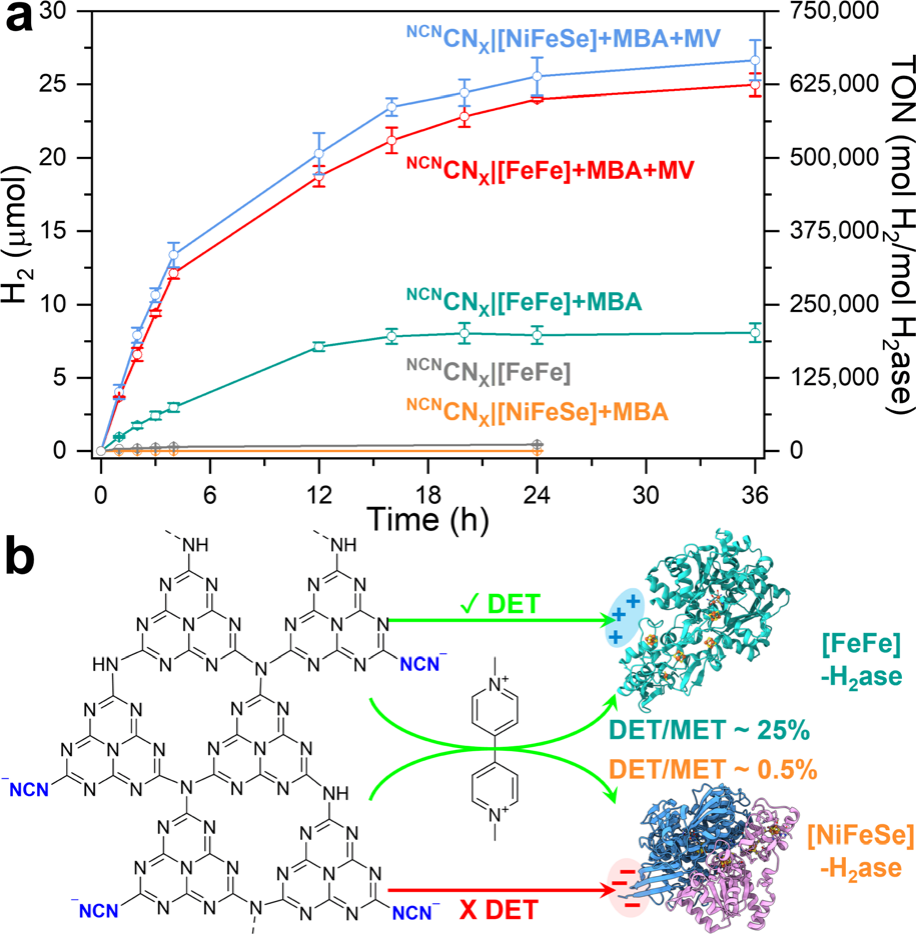
(a) Time-dependent photocatalytic H_2_ evolution of ^NCN^CN_*X*_ with H_2_ases for direct electron transfer (DET) and mediated electron transfer (MET). (b) Schematic of electron transfer process. Conditions: 1 mL anaerobic MOPS buffer (0.1 M, pH 7) containing 50 mM 4-MBA, 2 mg ^NCN^CN_*X*_, 40 pmol H_2_ase (either *CpI* [FeFe] (PDB: 4XDC) or *Dv*H [NiFeSe] (PDB: 5JSH)), AM 1.5G irradiation, 600 rpm stirring, 25 °C. For MET experiments, 2 mM methyl viologen (MV) was used. Error bars represent the standard deviation for a sample size of 3.

Time-dependent photocatalytic H_2_ evolution using ^NCN^CN_*X*_|[FeFe]-H_2_ase complexes is illustrated in Fig. 2a. The TON is determined by the ratio between the number of moles of product (H_2_) and the number of moles of catalyst (H_2_ase) and the TOF is calculated by the TON per hour. Notably, a nearly linear increase in H_2_ yield is observed during the initial 4 h, reaching 3.0 ± 0.3 μmol with a TOF of 18 669 h^−1^. This TOF value is approximately 4.5 times higher than the previous benchmark (4117 h^−1^)^15^ and is even comparable to systems utilizing MET such as g-C_3_N_4_|TiO_2_ (TOF = 20 000 h^−1^)^16^ (note that previous systems used *Desulfomicrobium baculatum* (*Dmb*) [NiFeSe]-H_2_ases). Continuous irradiation of the ^NCN^CN_X_|[FeFe]-H_2_ase assemblies for 24 h yielded 7.9 ± 0.6 μmol of H_2_ (TON = 198 125). The efficient DET between ^NCN^CN_*X*_ and [FeFe]-H_2_ase can be attributed to the specific electrostatic interaction at the interface, which will be further evaluated by QCM and PEIS. Furthermore, 4-MBA was selectively oxidized to *p*-tolualdehyde (Fig. S8–S10),† with a H_2_ : *p*-tolualdehyde ratio of 0.77. The observed ratio indicates the deep trapping of some photogenerated electrons within the CN_*X*_ polymeric structure in addition to some buffer (MOPS) oxidation (see below).

Recent transient spectroscopic and electron paramagnetic resonance (EPR) studies extensively characterized the deep traps stored in ^NCN^CN_*X*_ on the time scale from ps to s.^23–26^ EPR analysis showed that these long-lived and deeply trapped photoelectrons emerged as blue radicals, processing a symmetric Gaussian line near the free electron *g* value at X-band frequency of ∼9.6 GHz.^24^ These blue radicals can also be visualized by UV-vis spectroscopy (Fig. S5†) and qualitatively by the eye (Fig. S11†). The oxidation ability of ^NCN^CN_*X*_ has been further evaluated using glycerol (a waste product from the biodiesel industry) as the reductant on a model ^NCN^CN_*X*_|Pt (2 wt%) system. The oxidation products are quantified as glyceraldehyde at 134.2 ± 5.7 μmol h^−1^ g^−1^ and dihydroxyacetone at 54.9 ± 6.8 μmol h^−1^ g^−1^ (Fig. S12–S15)† and the H_2_ yield is 94 ± 9 μmol h^−1^ g^−1^. Replacing 4-MBA with ethylenediaminetetraacetic acid (EDTA) led to a ∼16% enhancement of H_2_ yield (4 h) with ^NCN^CN_*X*_|[FeFe]-H_2_ase assemblies (Table S1†). This indicates that, under DET conditions, the rate of 4-MBA oxidation is limiting compared to HER. Despite this observed higher activity, it is worth noting that EDTA is considered as a sacrificial electron donor, and its oxidation results in a range of products that cannot be easily characterized. As a result, the primary focus of this study is the conversion of 4-MBA to *p*-tolualdehyde, serving as a model reaction for the selective oxidation of alcohols to aldehydes.

Exclusion controls were conducted by removing individual components from the photocatalytic system (Table S1†). As depicted in Fig. 2a and S16,† the system exhibited significantly reduced efficiency in the absence of any component, with H_2_ yields below 0.3 μmol over 4 h. Minor H_2_ evolution activity was observed with ^NCN^CN_*X*_|[FeFe]-H_2_ase assemblies even in the absence of 4-MBA, yielding 285 ± 24 nmol H_2_ in 4 h. This observation suggests that MOPS serves as a much less efficient electron donor in the photocatalytic reaction. NMR analysis (Fig. S17–S20†) provides evidence of MOPS oxidation during photocatalysis. However, it is noteworthy that alcohol oxidation on ^NCN^CN_*X*_ is so efficient and selective that the photocatalytic activity of MOPS oxidation is only ∼5% compared to 4-MBA oxidation in 24 h (Fig. 2a). Upon replacing MOPS buffer with pH 7 phosphate buffer, the reaction is drastically reduced in the absence of 4-MBA, accompanied by a ∼27% decrease in photocatalytic activity in the presence of 4-MBA (Fig. S21, Table S2†). This observation is in line with literature that MOPS as a standard Good's buffer can maintain high *in vitro* biochemical and biological activities.^27^ Notably, control experiments were also performed to validate the proposed electrostatic interactions using a *Dv*H [NiFeSe]-H_2_ase, differing from the previously reported *Dmb* [NiFeSe]-H_2_ase.^15,16^ The distal FeS cluster of both [NiFeSe]-H_2_ases near its surface is surrounded by amino acids that lead to a local negative charge, serving as the electron entry point for interaction with the positively charged heme of cytochrome *c*_3_ during electron transfer *in vivo*.^28^ Interfacing *Dv*H [NiFeSe]-H_2_ase with ^NCN^CN_*X*_ resulted in the production of 8.6 ± 0.33 nmol of H_2_ in 4 h, with a significantly lower TON of 215 (Fig. 2a). These results indicate that electrostatic repulsion prevents DET in this system. A detailed comparison among state-of-the-art photocatalytic systems combining carbonaceous photocatalysts and H_2_ase are listed in Table 1.

State-of-the-art photocatalytic systems combining carbonaceous light absorbers and H_2_ases

**Table:** 

Photocatalytic system (DET)	TOF^a^ (h^−1^)	TON^b^	AQE	Ref.
^NCN^CN_*X*_|[FeFe] + MBA^d^	18 669	198 125	0.35%	This work
g-C_3_N_4_|[NiFeSe] + EDTA^d^	4117	36 000	0.07%	15
CDs|[NiFeSe] + EDTA^d^	3125	44 000	0.36%	29
CDs|[FeFe] + TEOA^e^	1500	19 000	1.7%	14

a TOF is calculated from 4 h photocatalysis.

b TON is calculated from 24 h photocatalysis.

c TiO_2_ as a non-diffusional electron mediator. Illumination conditions.

d AM 1.5G, 100 mW cm^−2^, Xe lamp.

e 50 mW cm^−2^, LED lamp.

**Table:** 

Photocatalytic system (MET)	TOF^a^ (h^−1^)	TON^b^	AQE	Ref.
^NCN^CN_*X*_|[FeFe] + MBA + MV^d^	75 769	600 350	1.4%	This work
^NCN^CN_*X*_|[NiFeSe] + MBA + MV^d^	83 588	638 825	N.A.	This work
g-C_3_N_4_|TiO_2_|[NiFeSe] + EDTA^c^^,^^d^	20 000	275 000	0.51%	16
CDs|[FeFe] + TEOA + MV^e^	2000	32 000	N.A.	14

To determine the charge transfer efficiency of DET, methyl viologen (MV) as a soluble electron mediator is used to activate MET (Fig. 2a). Note that the presence of MV may suppress DET due to the kinetic and thermodynamic favorable one electron reduction of MV molecules to MV radicals.^25,30^ Upon the addition of MV (2 mM), the H_2_ yield reaches 24.0 ± 0.1 μmol for *CpI* [FeFe]-H_2_ase and 25.6 ± 1.3 μmol for *Dv*H [NiFeSe]-H_2_ase after 24 h of irradiation. The comparable H_2_ yields, despite differences in specific activity, indicate that the rate limiting step during MET is not enzyme turnover but the photoactivity of ^NCN^CN_*X*_, as evidenced by control experiments (Table S1†). Specifically, by doubling the H_2_ase loading from 40 pmol to 80 pmol, no significant changes in H_2_ yields were observed over both 4 hour and 24 hour periods under MET conditions. The efficiency of DET is qualitatively determined by the DET : MET ratio, defined by the ratio of H_2_ yield in the absence of MV (DET) and in the presence of MV (MET). In the case of [FeFe]-H_2_ase and [NiFeSe]-H_2_ase, DET : MET ratios of approximately 25% and 0.5% are observed, respectively (Fig. S22†). By comparing the results obtained from DET and MET, a schematic representation can be depicted in Fig. 2b. It highlights the establishment of efficient electron transfer directly between ^NCN^CN_*X*_ and [FeFe]-H_2_ase, with a DET : MET ratio of 25%. This finding emphasizes the effectiveness and benefits of electrostatic interactions in facilitating DET. Conversely, the electrostatic repulsion between [NiFeSe]-H_2_ase and ^NCN^CN_*X*_ prevents DET, resulting in a low DET : MET ratio of 0.5%. Notably, the apparent quantum efficiency (AQE) measured at 450 nm with ^CN^CN_*X*_|[FeFe]-H_2_ase assemblies under DET and MET conditions are 0.35% and 1.4% (Table S3†), respectively. In contrast, a model ^NCN^CN_*X*_|Pt (2 wt%) system yields 0.28 ± 0.05 mmol h^−1^ g^−1^ H_2_ and 0.51 ± 0.09 mmol h^−1^ g^−1^*p*-tolualdehyde with an AQE of 1.92% at 450 nm. In terms of the overall stability of the designed systems, the DET system exhibited a rather linear photocatalytic activity up to 12 h. While MET systems are fully inactive only after 20 h. Long-term experiments up to 36 h confirmed these trends, with no further H_2_ production in DET after 24 h and minimal H_2_ yield increases in MET (24.0 to 25.0 μmol for [FeFe] and 25.6 to 26.7 μmol for [NiFeSe]). These findings align with recent observations on carbon dot|[FeFe]-H_2_ase photocatalytic systems.^14^

**Fig. 3:**
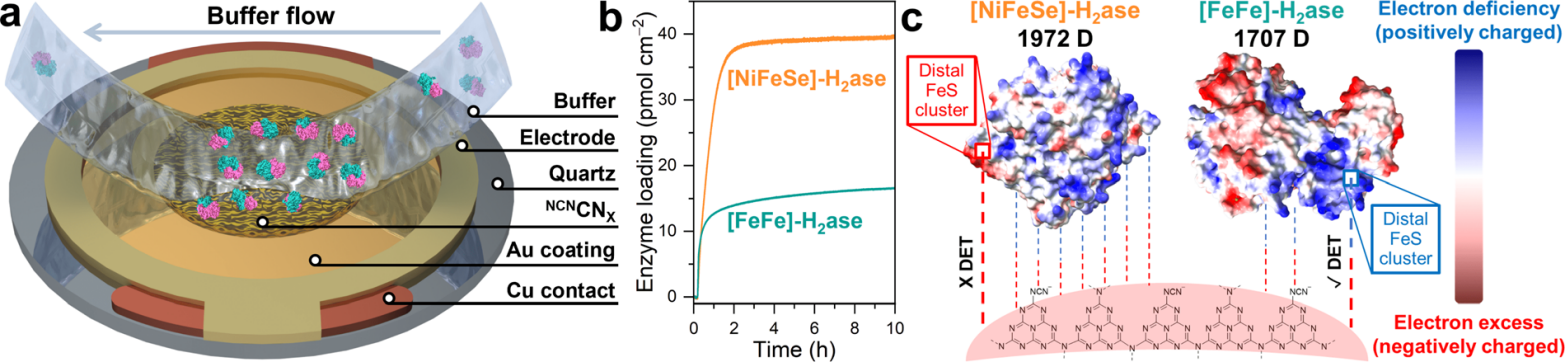
(a) Schematic illustration of a ^NCN^CN_*X*_-coated quartz chip. Buffer containing [FeFe]-H_2_ase (PDB: 4XDC) is flowing towards the chip and [FeFe]-H_2_ase is adsorbed onto the surface. (b) QCM analysis of the adsorption process of H_2_ase on a ^NCN^CN_*X*_-coated quartz chip. Conditions: 2 mL anaerobic MOPS buffer (0.1 M, pH 7) containing 50 mM 4-MBA, 80 pmol H_2_ase (either *CpI* [FeFe] or *Dv*H [NiFeSe]), 25 °C. (c) Electrostatic potential maps of *CpI* [FeFe] (PDB: 4XDC) and *Dv*H [NiFeSe] (PDB: 5JSH), and their interactions with ^NCN^CN_*X*_.

To gain deep insights into the interaction between ^NCN^CN_*X*_ and H_2_ase, QCM analysis was conducted. As shown in the schematic in Fig. 3a, the Au-coated quartz chip was functionalized with a thin layer of ^NCN^CN_*X*_ by drop casting 0.5 mL of an ultrasonicated suspension (0.1 mg mL^−1^) of ^NCN^CN_*X*_, to mimic the operando conditions during photocatalysis. By flowing a buffer solution containing enzymes on the chip, the adsorption process of H_2_ase at the surface of ^NCN^CN_*X*_ can be monitored as a function of time and quantified based on the Sauerbrey equation.^31^Fig. 3b shows the QCM analysis of *CpI* [FeFe]-H_2_ase and *Dv*H [NiFeSe]-H_2_ase on the ^NCN^CN_*X*_-modified chip. After establishing a stable baseline by circulating 0.1 M MOPS pH 7 buffer with 50 mM 4-MBA, the enzymes were introduced separately at the same concentration as in the photocatalysis experiments. The adsorption of both H_2_ases on ^NCN^CN_*X*_ exhibits in two distinct stages, a fast adsorption process before 1.5 h and slow adsorption after 1.5 h. Interestingly, a higher amount of [NiFeSe]-H_2_ase (39.5 pmol cm^−2^) is adsorbed onto ^NCN^CN_*X*_ compared to [FeFe]-H_2_ase (16.6 pmol cm^−2^) over 10 h. The observed adsorption profiles can be explained by the proposed electrostatic interactions in Fig. 3c. Based on the electrostatic potential maps (Fig. 3c), both H_2_ases exhibit distinct surface charge distribution. By indexing the specific protein structures, *CpI* [FeFe]-H_2_ase (PDB: 4XDC), and *DvH* [NiFeSe]-H_2_ase (PDB: 5JSH), within the protein dipole moment database,^32^ it is found that [NiFeSe]-H_2_ase possesses a larger dipole moment of 1972 *D* compared to [FeFe]-H_2_ase (1707 *D*), resulting in a stronger association (Fig. 3b). However, for [NiFeSe]-H_2_ase, DET can only be established *via* the negatively charged patch near the distal FeS cluster, which is unfavorable for the negatively charged ^NCN^CN_*X*_ and thus dramatically reduces DET to the enzyme active site for catalysis. Consequently, the strong association observed at the ^NCN^CN_*X*_|[NiFeSe]-H_2_ase interface is non-specific and results mainly in inactive biohybrid assemblies. In contrast, absorbed [FeFe]-H_2_ase has positively charged distal FeS cluster that can specifically interact with negatively charged ^NCN^CN_*X*_ for DET.^22,28^ The presence of other positively charged regions in the [FeFe]-H_2_ase (Fig. 3c) might also attract ^NCN^CN_*X*_. However, due to the rigidity of the heptazine-based ^NCN^CN_*X*_ (A–B′ stacking), proper orientation for DET near the distal FeS cluster could be hindered. This might explain the observed relatively low 25% DET/MET ratio. Therefore, QCM analysis provides valuable insights into the significance of specific interactions in facilitating DET.^33^

**Fig. 4:**
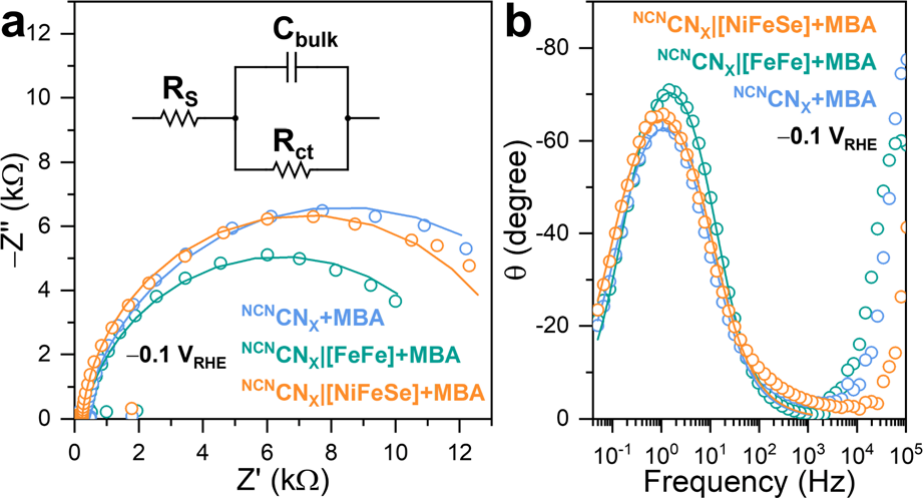
(a) Nyquist plots and (b) Bode phase plots of PEIS signal (open circles) with corresponding fitting curves (solid lines). Inset: proposed equivalent circuit to fit the impedance response. Conditions: 20 mL anaerobic MOPS buffer (0.1 M, pH 7) containing 50 mM 4-MBA, Ag/AgCl (3 M NaCl) reference electrode, Pt mesh counter electrode, AM 1.5G irradiation, 25 °C.

To investigate the charge carrier dynamics between H_2_ases and ^NCN^CN_*X*_, PEIS was performed using a three-electrode configuration. By applying a sinusoidal potential modulation to the ^NCN^CN_*X*_-modified working electrode, which was made by depositing a ^NCN^CN_*X*_ suspension (5 μL, 24 mg mL^−1^) on FTO-coated glass,^23^ the impedance was recorded as the ratio of the complex-valued potential and current.^34^ A Randles circuit consisting of a series resistance (*R*_S_) in parallel with a combination of bulk capacitance (*C*_bulk_) and charge transfer resistance (*R*_ct_), was proposed to fit the impedance response (Fig. 4a).^35^ The Nyquist plot (Fig. 4a) of the impedance response measured at −0.1 V *vs.* the reversible hydrogen electrode (RHE), is dominated by a single semicircle with no indication of a Warburg diffusion element. The proposed equivalent circuit provided a good fit (*r*^2^ > 0.95) to the impedance response, enabling quantitative analysis of the charge transfer process.

Upon introduction of [FeFe]-H_2_ase on the working electrode, a decrease in the semicircle diameter is observed, corresponding to a decrease in *R*_ct_ from 15 975 Ω to 12 317 Ω. This indicates that [FeFe]-H_2_ase, as a biocatalyst, facilitates the charge transfer from ^NCN^CN_*X*_ to the electrolyte for HER. Likewise, ^NCN^CN_*X*_|[NiFeSe]-H_2_ase shows a *R*_ct_ of 13 950 Ω, similar to the bare ^NCN^CN_*X*_. Such behaviors have been widely observed when incorporating synthetic co-catalysts onto semiconductors.^36^ The fitting results allowed determination of the pseudo first-order rate constant for charge transfer (*k*_ct_), based on the phenomenological model developed for an illuminated photoelectrode.^37,38^ Specifically, the angular frequency at the maximum imaginary component of the semicircle in Nyquist plot (Fig. 4a) is equal to *k*_ct_. The addition of [FeFe]-H_2_ase significantly enhances *k*_ct_ from 6.85 s^−1^ to 11.99 s^−1^, whereas [NiFeSe]-H_2_ase shows a negative impact on *k*_ct_ with a value of 5.02 s^−1^, further confirming the importance of specific interactions in facilitating the charge transfer process. The Bode phase plots (Fig. 4b) revealed that the charge transfer process occurred within the frequency range of 0.1 Hz to 1 kHz, consistent with the reported timeframe for photocatalytic HER using CN_*X*_.^36^ Within this range, the characteristic frequency at the maximum phase shift of ^NCN^CN_*X*_|[FeFe]-H_2_ase are higher than ^NCN^CN_*X*_|[NiFeSe]-H_2_ase and pristine ^NCN^CN_*X*_, indicating [FeFe]-H_2_ase can initiate a faster charge transfer process for HER. Likewise, the characteristic frequencies of ^NCN^CN_*X*_|[NiFeSe]-H_2_ase and pristine ^NCN^CN_*X*_ remain the same, meaning that DET cannot be established between ^NCN^CN_*X*_ and [NiFeSe]-H_2_ase. The voltage-dependent impedance response is illustrated in Fig. S23.† A more negative applied potential yields a diminished semicircular feature in the Nyquist plots, indicating reduced charge transfer resistance. This observation arises from the introduction of a larger band bending, resulting in improved separation of photogenerated charges.^39,40^ Consequently, a greater population of free charge carriers is localized within the semiconductor, increasing the conductivity of ^NCN^CN_*X*_. Notably, the RC response of the conductive substrate forms a semicircle with a diameter of approximately 200 Ω in the Nyquist plots (Fig. S24a†) at high frequency region (10 kHz to 1 MHz, Fig. S24b†) in the Bode phase plots.^23^ This impedance study on CN_*X*_ with H_2_ase demonstrates that a specific interaction enables efficient DET by decreasing in *R*_ct_ and increasing in *k*_ct_.

The charge carrier dynamics between H_2_ases and ^NCN^CN_*X*_ were further examined using IMVS and TPC techniques in a three-electrode setup. IMVS is a spectroelectrochemical method widely employed in assessing electron recombination processes in photovoltaics. It monitors the open circuit voltage response to the sinusoidally modulated incident light intensity. The characteristic frequency observed at the minimum point of the Nyquist plot (*f*_min_) directly correlates to the time constant of electron recombination. This parameter can be calculated using the following equation, providing the first-order electron lifetime *τ*_*n*_:^41^*τ*_*n*_ = (2π*f*_min_)^−1^

**Fig. 5:**
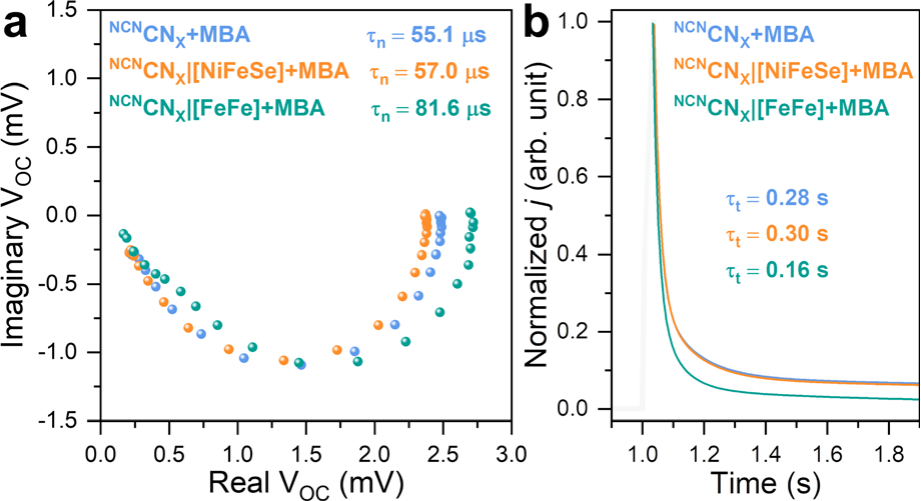
(a) Nyquist plots of IMVS response. (b) Normalized TPC response with corresponding exponential fitting curves. Conditions: 20 mL anaerobic MOPS buffer (0.1 M, pH 7) containing 50 mM 4-MBA, Ag/AgCl (3 M NaCl) reference electrode, Pt mesh counter electrode, AM 1.5G irradiation, 25 °C.

This model has been recently expanded to photoanodes for solar water oxidation.^42,43^ Although IMVS is not *operando* due to the distinct differences between a photoelectrode and a photocatalyst, photoelectrochemical techniques have been widely employed to gain insights into charge carrier behaviors in photochemical systems. This equation can be applied to a ^NCN^CN_*X*_-based photoelectrode due to it functions as a photoanode in the presence of 4-MBA and under open circuit conditions.^23^ As depicted in Fig. 5a, the IMVS response exhibits a distinct semicircle in quadrant IV of the Nyquist plot, suggesting rapid kinetics in 4-MBA oxidation, similar to the cases of sacrificial Na_2_SO_3_ and H_2_O_2_ oxidation on a hematite photoanode.^42,43^

The non-specific interaction between [NiFeSe]-H_2_ase and ^NCN^CN_*X*_ yields *τ*_*n*_ of 57.0 μs, whereas 55.1 μs is observed for pristine ^NCN^CN_*X*_. This consistency indicates that the charge recombination process remains unaffected, matching with our previous observations of the absence of DET between [NiFeSe]-H_2_ase and ^NCN^CN_*X*_. Upon the introduction of [FeFe]-H_2_ase onto ^NCN^CN_*X*_, we observe a prolonged *τ*_*n*_ of 81.6 μs that can be explained as follows: Even under open circuit conditions, where no net electron exchange occurs at the ^NCN^CN_*X*_|electrolyte interface, photogenerated electrons theoretically have the potential to react with protons *via* H_2_ases. Note that a TOF of 18 669 h^−1^ (^NCN^CN_*X*_|[FeFe]-H_2_ase) corresponds to a frequency of 5.2 Hz. Therefore, all losses of photogenerated electrons within the measured frequency range (0.5 MHz to 0.5 Hz) are a combination of bulk recombination and catalytic reaction. The influence of a catalytic overlayer on IMVS response remains a topic of debate. Recent studies indicate that a co-catalyst overlayer can delocalize photogenerated charge carriers from the bulk photoelectrode, promoting charge separation and prolonging electron lifetime.^42^ Here, despite the absence of net exchange current, we hypothesize that photogenerated electrons can be stored in H_2_ase in the form of metal hydrides and reduced FeS clusters,^44^*i.e.*, reversible intermediates for H_2_ evolution reaction. This storage mechanism reduces the probability of charge recombination with holes, resulting in an extended electron lifetime. IMVS observations are in line with the recent transient spectroscopic study on the impact of electron accumulation to charge recombination in ^NCN^CN_*X*_.^25^ Thus, we demonstrate the use of IMVS on carbon nitride materials and on studying bio-hybrids.

Having gained insights into charge recombination, we conducted TPC measurements to assess the influence of H_2_ases on the electron extraction process of ^NCN^CN_*X*_. The normalized TPC response, illustrated in Fig. 5b, reveals that the integration of [FeFe]-H_2_ase with ^NCN^CN_*X*_ leads to a significant reduction in electron transit time (*τ*_*t*_) from 0.28 s to 0.16 s. This indicates that [FeFe]-H_2_ase, acting as a co-catalyst for ^NCN^CN_*X*_, can effectively collect photoelectrons for HER, thereby facilitating electron transport within ^NCN^CN_*X*_.^45,46^ Likewise, ^NCN^CN_*X*_|[NiFeSe]-H_2_ase assemblies are non-specific, resulting in an unchanged *τ*_*t*_ of 0.30 s. The combined results from IMVS and TPC highlight the favorable effects of DET between [FeFe]-H_2_ase and ^NCN^CN_*X*_ on both charge recombination and transport. In contrast, the non-specifically interacted ^NCN^CN_*X*_|[NiFeSe]-H_2_ase assemblies demonstrate minimal alterations in both *τ*_*n*_ and *τ*_*t*_.

## Conclusions

We present an electrostatic strategy for linking enzymes with carbon nitrides, demonstrating a benchmark for DET between ^NCN^CN_*X*_ and [FeFe]-H_2_ase for solar H_2_ production with a TON of 2 × 10^5^ and a DET/MET ratio of 25% over 24 h. In contrast, the electrostatic repulsion between [NiFeSe]-H_2_ase and ^NCN^CN_*X*_ drastically reduced DET, leading to a DET/MET ratio of 0.5%. QCM analysis demonstrates that specific interactions play a pivotal role in enabling DET, irrespective of the observed differences in the adsorption profiles. Complementary spectroelectrochemical analysis using PEIS, IMVS, and TPC show that interfacing [FeFe]-H_2_ase with ^NCN^CN_*X*_ facilitates charge transfer and suppresses charge recombination, as evidenced by a 23% less resistive *R*_ct_, a 75% faster *k*_ct_, a 48% longer *τ*_*n*_, and a 43% shorter *τ*_*t*_ than bare ^NCN^CN_*X*_. This study provides a promising and straightforward approach for achieving efficient electron transfer between carbon nitride and enzymes and serves as a reference for studying the charge carrier behavior of enzyme-photocatalyst assemblies using interfacial characterizations.

## Data availability

Data supporting the findings of this study are available from the Cambridge data repository: https://doi.org/10.17863/CAM.106936.

## Author contributions

Yongpeng Liu conceptualization, data curation, software, formal analysis, funding acquisition, investigation, visualization, methodology, writing – original draft, project administration, writing – review & editing; Carolina Pulignani conceptualization, resources, data curation, formal analysis, investigation, visualization, methodology, writing – original draft, writing – review & editing; Sophie Webb resources, investigation, methodology, writing – review & editing; Samuel J. Cobb data curation, investigation, methodology, writing – review & editing; Santiago Rodríguez-Jiménez formal analysis, investigation, methodology, writing – review & editing; Dongseok Kim investigation, methodology; Ross D. Milton conceptualization, resources, supervision, funding acquisition, validation, writing – original draft, project administration, writing – review & editing; Erwin Reisner conceptualization, resources, formal analysis, supervision, funding acquisition, validation, investigation, visualization, writing – original draft, project administration, writing – review & editing.

## Conflicts of interest

There are no conflicts to declare.

## Supplementary Material

SC-015-D4SC00640B-s001

## References

[cit1] Nandy S., Savant S. A., Haussener S. (2021). Chem. Sci..

[cit2] Wang X., Maeda K., Thomas A., Takanabe K., Xin G., Carlsson J. M., Domen K., Antonietti M. (2009). Nat. Mater..

[cit3] Kwak M., Bok J., Lee B.-H., Kim J., Seo Y., Kim S., Choi H., Ko W., Antink W. H., Lee C. W., Yim G. H., Seung H., Park C., Lee K.-S., Kim D.-H., Hyeon T., Yoo D. (2022). Chem. Sci..

[cit4] Lau V. W., Moudrakovski I., Botari T., Weinberger S., Mesch M. B., Duppel V., Senker J., Blum V., Lotsch B. V. (2016). Nat. Commun..

[cit5] Kasap H., Caputo C. A., Martindale B. C. M., Godin R., Lau V. W., Lotsch B. V., Durrant J. R., Reisner E. (2016). J. Am. Chem. Soc..

[cit6] Lubitz W., Ogata H., Rüdiger O., Reijerse E. (2014). Chem. Rev..

[cit7] Le Goff A., Artero V., Jousselme B., Tran P. D., Guillet N., Métayé R., Fihri A., Palacin S., Fontecave M. (2009). Science.

[cit8] Lubitz W., Reijerse E., van Gastel M. (2007). Chem. Rev..

[cit9] Si G., Wang W.-G., Wang H.-Y., Tung C.-H., Wu L.-Z. (2008). Inorg. Chem..

[cit10] Jian J.-X., Ye C., Wang X.-Z., Wen M., Li Z.-J., Li X.-B., Chen B., Tung C.-H., Wu L.-Z. (2016). Energy Environ. Sci..

[cit11] Fang X., Kalathil S., Reisner E. (2020). Chem. Soc. Rev..

[cit12] Kornienko N., Zhang J. Z., Sakimoto K. K., Yang P., Reisner E. (2018). Nat. Nanotechnol..

[cit13] Brown K. A., Dayal S., Ai X., Rumbles G., King P. W. (2010). J. Am. Chem. Soc..

[cit14] Holá K., Pavliuk M. V., Németh B., Huang P., Zdražil L., Land H., Berggren G., Tian H. (2020). ACS Catal..

[cit15] Caputo C. A., Gross M. A., Lau V. W., Cavazza C., Lotsch B. V., Reisner E. (2014). Angew. Chem., Int. Ed..

[cit16] Caputo C. A., Wang L., Beranek R., Reisner E. (2015). Chem. Sci..

[cit17] Zhang Y., Liu J. (2022). Chem.–Eur. J..

[cit18] Zhang S., Zhang Y., Chen Y., Yang D., Li S., Wu Y., Sun Y., Cheng Y., Shi J., Jiang Z. (2021). ACS Catal..

[cit19] Liu J., Liu Y., Liu N., Han Y., Zhang X., Huang H., Lifshitz Y., Lee S.-T., Zhong J., Kang Z. (2015). Science.

[cit20] Liu Y., Webb S., Moreno-García P., Kulkarni A., Maroni P., Broekmann P., Milton R. D. (2023). JACS Au.

[cit21] Marques M. C., Tapia C., Gutiérrez-Sanz O., Ramos A. R., Keller K. L., Wall J. D., De Lacey A. L., Matias P. M., Pereira I. A. C. (2017). Nat. Chem. Biol..

[cit22] Peters J. W., Lanzilotta W. N., Lemon B. J., Seefeldt L. C. (1998). Science.

[cit23] Pulignani C., Mesa C. A., Hillman S. A. J., Uekert T., Giménez S., Durrant J. R., Reisner E. (2022). Angew. Chem., Int. Ed..

[cit24] Lau V. W., Klose D., Kasap H., Podjaski F., Pignié M.-C., Reisner E., Jeschke G., Lotsch B. V. (2017). Angew. Chem., Int. Ed..

[cit25] Yang W., Godin R., Kasap H., Moss B., Dong Y., Hillman S. A. J., Steier L., Reisner E., Durrant J. R. (2019). J. Am. Chem. Soc..

[cit26] Godin R., Wang Y., Zwijnenburg M. A., Tang J., Durrant J. R. (2017). J. Am. Chem. Soc..

[cit27] Good N. E., Winget G. D., Winter W., Connolly T. N., Izawa S., Singh R. M. M. (1966). Biochemistry.

[cit28] Matias P. M., Soares C. M., Saraiva L. M., Coelho R., Morais J., Le Gall J., Carrondo M. A. (2001). JBIC, J. Biol. Inorg. Chem..

[cit29] Hutton G. A. M., Reuillard B., Martindale B. C. M., Caputo C. A., Lockwood C. W. J., Butt J. N., Reisner E. (2016). J. Am. Chem. Soc..

[cit30] Heyrovský M. (1987). J. Chem. Soc. Chem. Commun..

[cit31] Sauerbrey G. (1959). Z. Med. Phys..

[cit32] Felder C. E., Prilusky J., Silman I., Sussman J. L. (2007). Nucleic Acids Res..

[cit33] Badiani V. M., Casadevall C., Miller M., Cobb S. J., Manuel R. R., Pereira I. A. C., Reisner E. (2022). J. Am. Chem. Soc..

[cit34] Bard A. J. and Faulkner L. R., Electrochemical Methods: Fundamentals and Applications, John Wiley & Sons, Incorporated, 2nd edn, 2000

[cit35] Randles J. E. B. (1947). Discuss. Faraday Soc..

[cit36] Jiang W., Zhao Y., Zong X., Nie H., Niu L., An L., Qu D., Wang X., Kang Z., Sun Z. (2021). Angew. Chem., Int. Ed..

[cit37] Wijayantha K. G. U., Saremi-Yarahmadi S., Peter L. M. (2011). Phys. Chem. Chem. Phys..

[cit38] Xu P., Gray C. L., Xiao L., Mallouk T. E. (2018). J. Am. Chem. Soc..

[cit39] Bisquert J. (2002). J. Phys. Chem. B.

[cit40] Liu Y., Quiñonero J., Yao L., Pereira X. D. C., Mensi M., Gómez R., Sivula K., Guijarro N. (2021). J. Mater. Chem. A.

[cit41] Krüger J., Plass R., Grätzel M., Cameron P. J., Peter L. M. (2003). J. Phys. Chem. B.

[cit42] Liu Y., Guijarro N., Sivula K. (2020). Helv. Chim. Acta.

[cit43] Thorne J. E., Jang J.-W., Liu E. Y., Wang D. (2016). Chem. Sci..

[cit44] Schilter D., Camara J. M., Huynh M. T., Hammes-Schiffer S., Rauchfuss T. B. (2016). Chem. Rev..

[cit45] Le Formal F., Sivula K., Grätzel M. (2012). J. Phys. Chem. C.

[cit46] Liu Y., Xia M., Yao L., Mensi M., Ren D., Grätzel M., Sivula K., Guijarro N. (2021). Adv. Funct. Mater..

