# Supplementary material for: Electrostatic [FeFe]-hydrogenase–carbon nitride assemblies for efficient solar hydrogen production
Source: Chem Sci. 2024 Mar 13;15(16):6088–94. doi: 10.1039/d4sc00640b (PMC11040649; doi:10.1039/d4sc00640b)
Supplement: SC-015-D4SC00640B-s001 [file SC-015-D4SC00640B-s001.pdf]

Electronic Supplementary Material (ESI) for Chemical Science.
This journal is © The Royal Society of Chemistry 2024

Chemical Science

Electronic Supplementary Information (ESI)

Electrostatic [FeFe]-hydrogenase–carbon nitride assemblies for efficient solar hydrogen production

Yongpeng Liu,[a†] Carolina Pulignani,[a†] Sophie Webb,[bc] Samuel J. Cobb,[a] Santiago Rodríguez-Jiménez,[a] Dongseok Kim,[a] Ross D. Milton[bc] and Erwin Reisner*[a]

[a] *Yusuf Hamied Department of Chemistry, University of Cambridge, Cambridge CB2 1EW, UK. E-mail: reisner@ch.cam.ac.uk*
[b] *Department of Inorganic and Analytical Chemistry, University of Geneva, Geneva 4 1211, Switzerland.*
[c] *National Centre of Competence in Research (NCCR) Catalysis, University of Geneva, Geneva 4 1211, Switzerland.*
† These authors contributed equally to this work.

Contents

S1 Experimental Section . . . S6
S1.1 Materials . . . S6
S1.2 Synthesis of Graphitic Carbon Nitride (g-C_3N_4) . . . S6
S1.3 Synthesis of Cyanamide-Functionalized Carbon Nitride ($^{NCN}CN_X$) . . . S7
S1.4 Scanning Electron Microscope (SEM) . . . S7
S1.5 Attenuated Total Reflectance Fourier Transform Infrared (ATR-FTIR) Spectroscopy . . . S7
S1.6 Fluorescence Spectroscopy . . . S7
S1.7 Ultraviolet–Visible (UV−vis) Spectroscopy . . . S7
S1.8 Zeta Potential . . . S8
S1.9 Photocatalysis . . . S8
S1.10 Proton Nuclear Magnetic Resonance (^1H NMR) Spectroscopy . . . S9
S1.11 Gas Chromatography (GC) . . . S9
S1.12 High-Performance Liquid Chromatography (HPLC) . . . S9
S1.13 Turnover Number (TON) and Turnover Frequency (TOF) . . . S10
S1.14 Apparent Quantum Efficiency (AQE) . . . S10
S1.15 Quartz Crystal Microbalance (QCM) . . . S10
S1.16 Photoelectrochemical Impedance Spectroscopy (PEIS) . . . S11
S1.17 Intensity-Modulated Photovoltage Spectroscopy (IMVS) . . . S12
S1.18 Transient Photocurrent Spectroscopy (TPC) . . . S12
S2 Comparison between a H_2ase and a H_2ase Mimic . . . S13
S3 Scanning Electron Microscope (SEM) Images . . . S14
S4 Attenuated Total Reflectance Fourier Transform Infrared (ATR-FTIR) Spectroscopy . . . S15
S5 Fluorescence Spectroscopy . . . S16

S6 Ultraviolet–Visible (UV−vis) Spectroscopy S17
S7 Zeta Potential Measurements S18
S8 ^{1}H NMR Spectroscopy for Evaluating 4-MBA Oxidation S20
S9 High-Performance Liquid Chromatography (HPLC) for 4-MBA Oxidation . . S21
S10 Photograph of Photoreactors Before and After Photocatalysis S23
S11 High-Performance Liquid Chromatography (HPLC) for Glycerol Oxidation . . S24
S12 Table of Photocatalytic H_2 Evolution under Different Conditions S28
S13 Exclusion Control Experiments for Photocatalytic H_2 Evolution S29
S14 ^{1}H NMR Spectroscopy for Evaluating MOPS Oxidation S30
S15 Photocatalytic H_2 Evolution with $^{NCN}CN_X$|[FeFe]-H_2ase Assemblies in MOPS and Phosphate S34
S16 Table of Photocatalytic H_2 Evolution with $^{NCN}CN_X$|[FeFe]-H_2ase Assemblies in MOPS and Phosphate S35
S17 Ratio of DET to MET S36
S18 Comparison among State-of-the-art Carbon Nitride Photocatalysts and [FeFe]-H_2ase Assemblies for H_2 Evolution S37
S19 Nyquist Plots of Impedance Response at Different Potentials S38
S20 High Frequency Impedance Response S39
References S40

List of Figures

S1 Comparison between a H_2ase and a H_2ase mimic S13
S2 SEM images of $^{NCN}CN_X$ S14
S3 FTIR spectrum of $^{NCN}CN_X$ S15
S4 Fluorescence spectrum of $^{NCN}CN_X$ S16
S5 UV−vis spectra of $^{NCN}CN_X$ (light) and $^{NCN}CN_X$ (dark) S17
S6 Zeta potential of $^{NCN}CN_X$ (light) and $^{NCN}CN_X$ (dark) S18
S7 Zeta potential of $^{NCN}CN_X$ as a function of pH S19
S8 ^{1}H NMR spectra of photocatalysis solution for 4-MBA oxidation at t=0 and t=4h S20
S9 HPLC spectrum of photocatalysis solution at t=24 h S21
S10 HPLC calibration curves for 4-MBA oxidation S22
S11 Photograph of photoreactors before and after photocatalysis S23
S12 HPLC spectrum of photocatalysis solution at t=2 h S24
S13 HPLC-UV (210 nm) spectrum of photocatalysis solution at t=2 h . . . S25
S14 HPLC-UV (270 nm) spectrum of photocatalysis solution at t=2 h . . . S26
S15 HPLC calibration curves for glycerol oxidation to glyceraldehyde and dihydroxyacetone S27
S16 Control experiments for photocatalytic H_2 evolution S29
S17 ^{1}H NMR spectra of photocatalysis solution before photocatalysis . . . S30
S18 ^{1}H NMR spectra of photocatalysis solution after photocatalysis S31
S19 Zoom up of ^{1}H NMR spectra of photocatalysis solution after photocatalysis S32
S20 Zoom up of ^{1}H NMR spectra of photocatalysis solution after photocatalysis S33
S21 Photocatalytic H_2 evolution in MOPS and phosphate S34
S22 Ratio of DET to MET S36

S23 Nyquist plots of impedance response at different potentials S38
S24 Nyquist plots and Bode phase plots of impedance response at high frequency region S39

List of Tables

S1 Table of photocatalytic H_2 evolution under different conditions S28
S2 Photocatalytic H_2 evolution in MOPS and phosphate S35
S3 Comparison among state-of-the-art carbon nitride photocatalysts and [FeFe]-H_2ase assemblies for H_2 evolution S37

S1 Experimental Section

S1.1 Materials

The chemicals and materials were purchased from commercial suppliers and used without further purification: N_2 gas bottle (2% CH_4 as internal standard, BOC), 3-(N-morpholino) propanesulfonic acid (MOPS, Sigma-Aldrich, ≥99.5%), MOPS sodium salt (Sigma-Aldrich, ≥99.5%), 4-methylbenzyl alcohol (4-MBA, Sigma-Aldrich, 98%), 4-methylbenzaldehyde (*p*-tolualdehyde, Thermo Scientific Chemicals, 99+%), potassium phosphate monobasic (KH_2PO_4, Sigma-Aldrich, ≥99.0%), potassium phosphate dibasic (K_2HPO_4, Sigma-Aldrich, ≥98%), ethylenediaminetetraacetic acid (EDTA, Sigma-Aldrich, 99.4-100.6%), EDTA disodium salt dihydrate (Sigma-Aldrich, 99.0-101.0%), chloroplatinic acid hydrate ($H_2PtCl_6 \cdot xH_2O$, Sigma-Aldrich, ≥99.9% trace metals basis), glycerol (Sigma-Aldrich, ≥99.0%), glyceraldehyde (Sigma-Aldrich, ≥90%), dihydroxyacetone (Sigma-Aldrich, ≥98%), melamine (Sigma-Aldrich, 99%), potassium thiocyanate (KSCN, Sigma-Aldrich, ≥99.0%), ethanol (Sigma-Aldrich, 96%), deuterium oxide (Sigma-Aldrich, 99.9 atom% D, contains 0.75 wt% 3-(trimethylsilyl) propionic-2,2,3,3-d_4 acid, sodium salt), fluorine doped tin oxide (FTO) coated glass slide (2 cm × 10 cm, Pilkington TEC 15, Xop Glass, 12 - 14 Ω/sq), and rubber septa (Subaseal). MilliQ H_2O (18.2 MΩ cm) was used for all the experiments. [FeFe]-H_2ase from *Clostridium pasteurianum* (*CpI*) and [NiFeSe]-H_2ase from *Desulfovibrio vulgaris* Hildenborough (*Dv*H) were expressed and purified based on published methods under anaerobic conditions.[S1,S2]

S1.2 Synthesis of Graphitic Carbon Nitride (g-C_3N_4)

Melamine (5 g) was placed in a ceramic crucible with a lid and heated up at 550 °C for three hours with a ramp rate of 1 °C min^{-1} under air, following a previously reported procedure.[S3] The g-C_3N_4 yellow powder (50% yield) was grounded with a mortar and pestle.

S1.3 Synthesis of Cyanamide-Functionalized Carbon Nitride ($^{NCN}CN_X$)

The grounded g-C_3N_4 powder was mixed with potassium thiocyanate in a 1:2 weight ratio. The mixture was placed in a ceramic boat and heated up first at 400 °C for 60 min and then at 500 °C for 30 min (ramp rate 30 °C min^{-1}) under Ar, following a published procedure.[S4] The $^{NCN}CN_X$ powder was allowed to cool down to room temperature, washed twice with water, once with water and ethanol (1:1 mixture), and dried overnight at room temperature.

S1.4 Scanning Electron Microscope (SEM)

Cyanamide-functionalized CN_X powder was placed on conductive carbon tape, the excess powder was removed and then sputter-coated with a 10 nm layer of Cr prior to measurement. SEM imgaes were aquired on a TESCAN MIRA3 FEG-SEM.

S1.5 Attenuated Total Reflectance Fourier Transform Infrared (ATR-FTIR) Spectroscopy

Attenuated total reflectance Fourier-transform infrared (ATR-FTIR) spectra were recorded on a Nicolet iS50 spectrometer.

S1.6 Fluorescence Spectroscopy

Fluorimetry was performed on an Edinburgh Instruments Spectrofluorometer FS5 equipped with Visible PMT-900 detector.

S1.7 Ultraviolet–Visible (UV−vis) Spectroscopy

UV−vis spectra were collected using a Cary 60 UV−vis spectrometer.

S1.8 Zeta Potential

Zeta potential was conducted by using a Malvern Zetasizer Nano ZS spectrometer equipped with a red laser (632.8 nm). For measuring the light-induced blue radical in $^{NCN}CN_X$, the zeta cell was first sealed in an anaerobic glovebox (MBraun, N_2 atmosphere, < 0.1 ppm O_2), then illuminated under AM 1.5G for 30 min until the color of suspension turns into blue.

S1.9 Photocatalysis

$^{NCN}CN_X$ (4 mg/mL) was dispersed in aqueous solution containing 50 mM 4-MBA and ultrasonicated for 10 min at 30 °C (Fisher Scientific, Fisherbrand™ Model 120 Sonic Dismembrator). The suspension was mixed with aqueous solution containing 0.2 M MOPS and 50 mM 4-MBA at 1:1 volume ratio to reach a final concentration of 2 mg/mL $^{NCN}CN_X$, 0.1 M MOPS, and 50 mM 4-MBA. 1 mL of the suspension was added in each photoreactor, after which H_2ase (either [FeFe] or [NiFeSe]) was added. All of the photoreactors were assembled and sealed in an anaerobic glovebox (MBraun, N_2 atmosphere, < 0.1 ppm O_2). During photocatalysis, the photoreactors were illuminated under simulated AM 1.5G (100 mW/cm^2, Newport xenon arc lamp housing 66921) and stirred at 600 rpm at 25 ^{o}C.

For photocatalysis experiments using inorganic buffer, a phosphate buffer containing KH_2PO_4 and K_2HPO_4 at pH 7 was used.

When 50 mM 4-MBA was replaced by EDTA, 100 mM EDTA was used.

When 50 mM 4-MBA was replaced by glycerol, 50 mM glycerol was used.

For photocatalysis experiments involving Pt, optimized Pt loading (2 wt %) was achieved by adding chloroplatinic acid hydrate as a precursor to form Pt nanoparticles onto carbon nitride via in situ photodeposition.[S5]

S1.10 Proton Nuclear Magnetic Resonance (^{1}H NMR) Spectroscopy

^{1}H NMR spectra were collected with a Bruker 400 MHz NMR spectrometer at 25 °C. Chemical shifts for ^{1}H NMR spectra are referenced relative to residual protons in the deuterated solvent (Eurisotop), and 3-(trimethylsilyl)propionic-2,2,3,3-d_4 acid, sodium salt, in D_2O was used as the internal standard (TSP).

S1.11 Gas Chromatography (GC)

The amount of H_2 produced was analyzed by headspace gas analysis using a Shimadzu Tracera GC-2010 Plus with a barrier dis-charge ionization detector. The GC-2010 Plus was equipped with a ShinCarbon micro ST column (0.53 mm diameter) kept at 40 °C using helium carrier gas. Aliquots of 100 μL of the headspace gas were removed from the sealed photocatalytic vials using a gastight syringe (Hamilton) for GC analysis.

S1.12 High-Performance Liquid Chromatography (HPLC)

A Waters Breeze system equipped with a refractive index (RID-2414) and diode array UV−vis (λ = 210 and 254 nm) detectors was employed to analyze the oxidation conversion and yield. More specifically, the 4-MBA (retention time 6.1 ± 0.2 min) and relative oxidation product, 4-methylbenzaldehyde (*p*-tolualdehyde, retention time 8.8 ± 0.2 min), were identified and quantified with a C18 column at 40 °C column temperature in isocratic flow mode (H_2O:MeCN, flow rate 0.5 mL min^{-1}). Calibration curves were conducted with external standards for both substrates.

For each HPLC measurement after photocatalysis, 100 μL of sample was diluted 10 times with 900 μL of H_2O in a 2 mL glass screw top vial (Thermo Fisher Scientific, Si/PTFE seal 8 mm) for injection.

S1.13 Turnover Number (TON) and Turnover Frequency (TOF)

The turnover number (TON) is determined by the ratio between the number of moles of product (H_2) and the number of moles of catalyst (H_2ase):

$$\text{TON} = \frac{n_{\text{product}}(\text{mol})}{n_{\text{catalyst}}(\text{mol})} = \frac{n_{\text{H}_2}(\text{mol})}{n_{\text{H}_2\text{ase}}(\text{mol})}$$

The turnover frequency (TOF) is determined by the TON per hour:

$$\text{TOF} = \frac{\text{TON}}{\text{hour}}$$

S1.14 Apparent Quantum Efficiency (AQE)

AQE measurements were conducted using a solar simulator (LOT-Quantum Design GmbH, LSN254) with a monochromator (LOT-Quantum Design GmbH, MSH300) at 450 nm wavelength. The AQE was calculated by:[S6]

$$\text{AQE} = \frac{\text{number of reacted electrons}}{\text{number of incident photons}} \times 100\% \tag{1}$$

$$= \frac{2\times \text{ H}_2 \text{ yield}}{\text{number of incident photons}} \times 100\% \tag{2}$$

S1.15 Quartz Crystal Microbalance (QCM)

QCM experiments were performed using a Biolin Q-Sense Explorer module and a custom-designed QCM electrochemical cell within an anaerobic glovebox (MBraun, N_2 atmosphere, < 0.1 ppm O_2). A gold-coated quartz chip with a surface area of 0.79 cm^2 was utilized. The chip was initially functionalized by drop casting 0.5 mL of an ultrasonicated suspension (0.1 mg/mL) of $^{NCN}CN_X$, forming a thin layer on the surface. To establish a stable baseline, prior to measurements, a solution of 2 mL H_2ase-free 0.1 M MOPS pH 7 buffer containing 50 mM 4-MBA was flowed through the system at a rate of 0.141 mL min^{-1} for a duration of 1 hour.

Once the baseline reached a steady state, 80 pmol of H_2ase (either [FeFe] or [NiFeSe]) was introduced into the 2 mL buffer solution. The adsorption of the enzyme onto the surface was quantified by monitoring changes in the resonance frequency of the piezoelectric quartz chip. To determine the corresponding mass change, the change in frequency (Δf) was analyzed using the Sauerbrey equation:[S7]

$$\Delta f = -\frac{2f_0^2}{A\sqrt{p_q \mu_q}}\Delta m$$

where f_0 is the resonance frequency (5 MHz) of the quartz oscillator, A is the piezoelectrically active crystal area, Δm is the change in mass, p_q is the density of quartz, and μ_q is the shear modulus of quartz. Assuming 25% of the adsorbed mass consisted of water molecules bound to the H_2ase, Δm can be converted into quantity of H_2ase.

S1.16 Photoelectrochemical Impedance Spectroscopy (PEIS)

PEIS measurements were carried out in an electrochemical cell with a 3-electrode configuration: a $^{NCN}CN_X$ working electrode, a Pt mesh counter electrode, and a RE-6 Ag/AgCl reference electrode (3 M NaCl gel, 0.55 mm diameter ceramic frit, MW-2030, BASi). 40 pmol H_2ases (either *CpI* [FeFe] or *DvH* [NiFeSe]) were drop-cast onto the working electrode. The 20 mL anaerobic electrolyte contains 0.1 M MOPS, pH 7, 50 mM 4-MBA. A 150 W xenon arc lamp (LOT-Quantum Design GmbH, LSE140/160.25C), that was calibrated to AM 1.5G, was used as light source. Impedance response was recorded with a potentiostat (IviumStat) with frequency ranges from 1 MHz to 50 mHz and a 25 mV sinusoidal amplitude. Impedance data was fitted with equivalent circuits using modeling software ZView2 (Scribner Associates).

The $^{NCN}CN_X$ working electrode was made by depositing $^{NCN}CN_X$ suspension on a FTO-coated glass, adapting a literature procedure.[S8] A 0.25 cm^2 Parafilm template, made with a drilling bill, was pressed onto the FTO side (1 cm $\times$ 2 cm) and slightly heated (45 s in a

120 °C drying oven) to ensure uniform adhesion of the mask to the slide. 6 mg of $^{NCN}CN_X$ was dispersed in 250 μL of ethanol and ultrasonicated for 30 min at 30 °C (Fisher Scientific, Fisherbrand™ Model 120 Sonic Dismembrator). 5 μL of the suspension was drop-casted onto the masked FTO glass and allowed to dry in air. The mask was removed and the samples were annealed for 1 h at 250 °C under Ar (ramp rate 10 °C min^{-1}).

S1.17 Intensity-Modulated Photovoltage Spectroscopy (IMVS)

IMVS measurements were carried out under open circuit condition in a single compartment electrochemical cell with a 2-electrode configuration containing a $^{NCN}CN_X$ working electrode and a RE-6 Ag/AgCl reference electrode (3 M NaCl gel, 0.55 mm diameter ceramic frit, MW-2030, BASi). 40 pmol H_2ases (either *CpI* [FeFe] or *DvH* [NiFeSe]) were drop-cast onto the working electrode. The 20 mL anaerobic electrolyte contains 0.1 M MOPS, pH 7, 50 mM 4-MBA. A 470 nm blue LED (TruOpto, OSUB5111P, 5 mm, 12000 mcd) was used as light source and was sinusoidally modulated (0.5 MHz to 0.5 Hz, ∼10% modulation depth) by a Bio-Logic VSP potentiostat. The open circuit voltage (V_{OC}) was recorded on a Bio-Logic VSP potentiostat.

S1.18 Transient Photocurrent Spectroscopy (TPC)

TPC measurements were conducted in a single compartment electrochemical cell with a 3-electrode configuration containing a $^{NCN}CN_X$ working electrode, a Pt mesh counter electrode, and a RE-6 Ag/AgCl reference electrode (3 M NaCl gel, 0.55 mm diameter ceramic frit, MW-2030, BASi). 40 pmol H_2ases (either *CpI* [FeFe] or *DvH* [NiFeSe]) were drop-cast onto the working electrode. The 20 mL anaerobic electrolyte contains 0.1 M MOPS, pH 7, 50 mM 4-MBA. An AM 1.5G solar light simulator (LOT-Quantum Design GmbH, LS0816-H/LSN558) with a built-in shutter was used as light source. TPC response was recorded at −0.1 V *vs* the reversible hydrogen electrode (RHE) on a Bio-Logic VSP potentiostat. TPC data was normalized and fitted with exponential decay function using OriginPro 2021b (OriginLab).

S2 Comparison between a H_2ase and a H_2ase Mimic

The $Fe_2S_2(CO)_6$ is a type of biomimetic catalyst that synthetically mimics the active site of the Fe_2S_2 subunit of the [FeFe]-H_2ase and there are significant differences between a small molecule catalyst and the natural macromolecular [FeFe]-H_2ase. As shown in Figure S1, a *CpI* [FeFe]-H_2ase contains 10,385 atoms (excluding H atom) with a molecular mass of 134.22 kDa (= 134,220 g/mol) and a spherical diameter around 10 nm,[S9] while a $Fe_2S_2(CO)_6$ molecular catalyst contains 16 atoms with a molecular mass of 0.344 kDa (= 344 g/mol) and a spherical diameter <1 nm.[S10] A *CpI* [FeFe]-H_2ase processes a complex electrostatic surface due to the coverage of various surface-exposed amino acids, and therefore establishing a strong and efficient electrostatic interaction is challenging to control in the field of semi-artificial photosynthesis.

CpI [FeFe]-H_2ase (PDB: 4XDC) **$Fe_2S_2(CO)_6$ [FeFe]-H_2ase mimic**

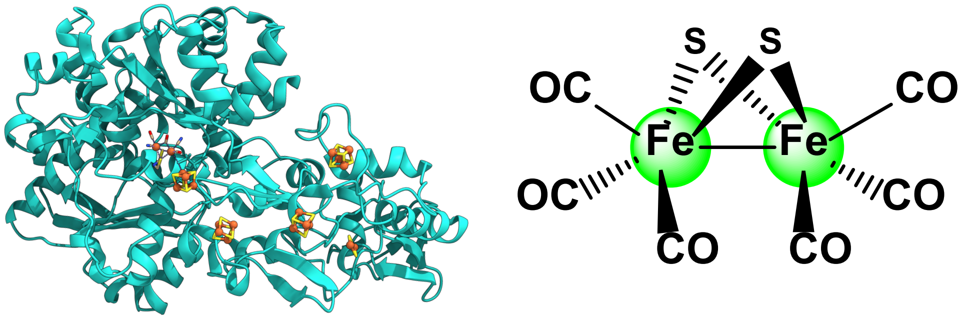

	CpI [FeFe]-H_2ase (PDB: 4XDC)	$Fe_2S_2(CO)_6$ [FeFe]-H_2ase mimic
**Atom count**	10,385 (excluding H)	16
**Molecular mass**	134.22 kDa (= 134,220 g/mol)	0.344 kDa (= 344 g/mol)
**Spherical diameter**	~10 nm	<1 nm

Figure S1: Comparison between a *CpI* [FeFe]-H_2ase (PDB: 4XDC) and a $Fe_2S_2(CO)_6$ [FeFe]-H_2ase mimic.

S3 Scanning Electron Microscope (SEM) Images

SEM images in Figure S2 show nanoparticle morphology of $^{NCN}CN_X$.

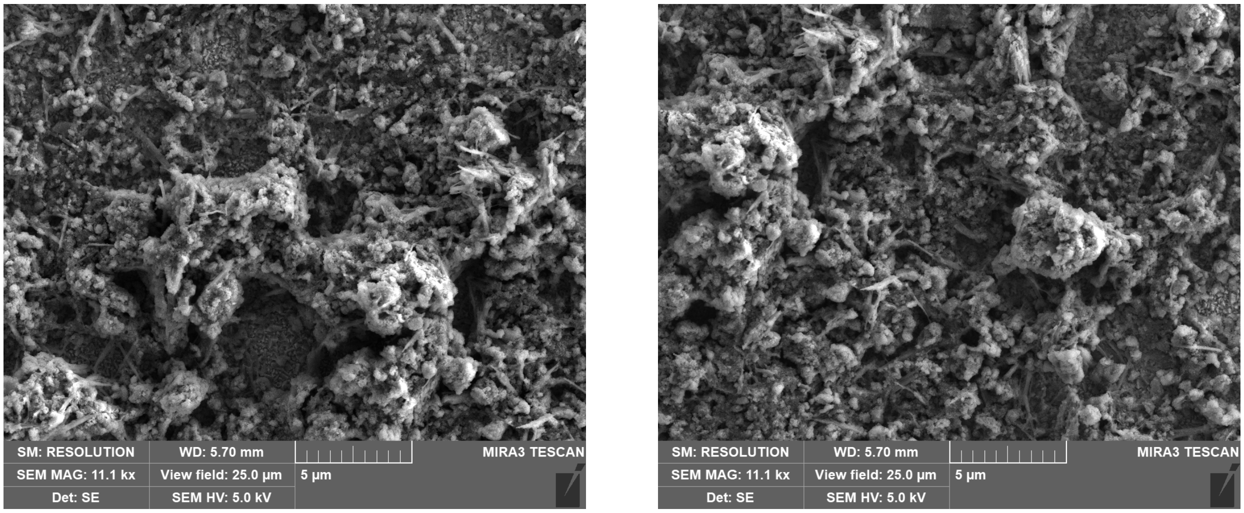

Figure S2: SEM images of $^{NCN}CN_X$.

S4 Attenuated Total Reflectance Fourier Transform Infrared (ATR-FTIR) Spectroscopy

Attenuated total reflectance Fourier transform infrared (ATR-FTIR) spectroscopy analysis confirms the characteristic bands of $^{NCN}CN_X$ in the following spectrum (Figure S3), incldluing C=N stretching and N-H bending (1645 cm^{-1}, 1578 cm^{-1}), C-N stretching (1425 cm^{-1}, 1382 cm^{-1}), and Heptazine ring stretching (804 cm^{-1}).[S4,S11]

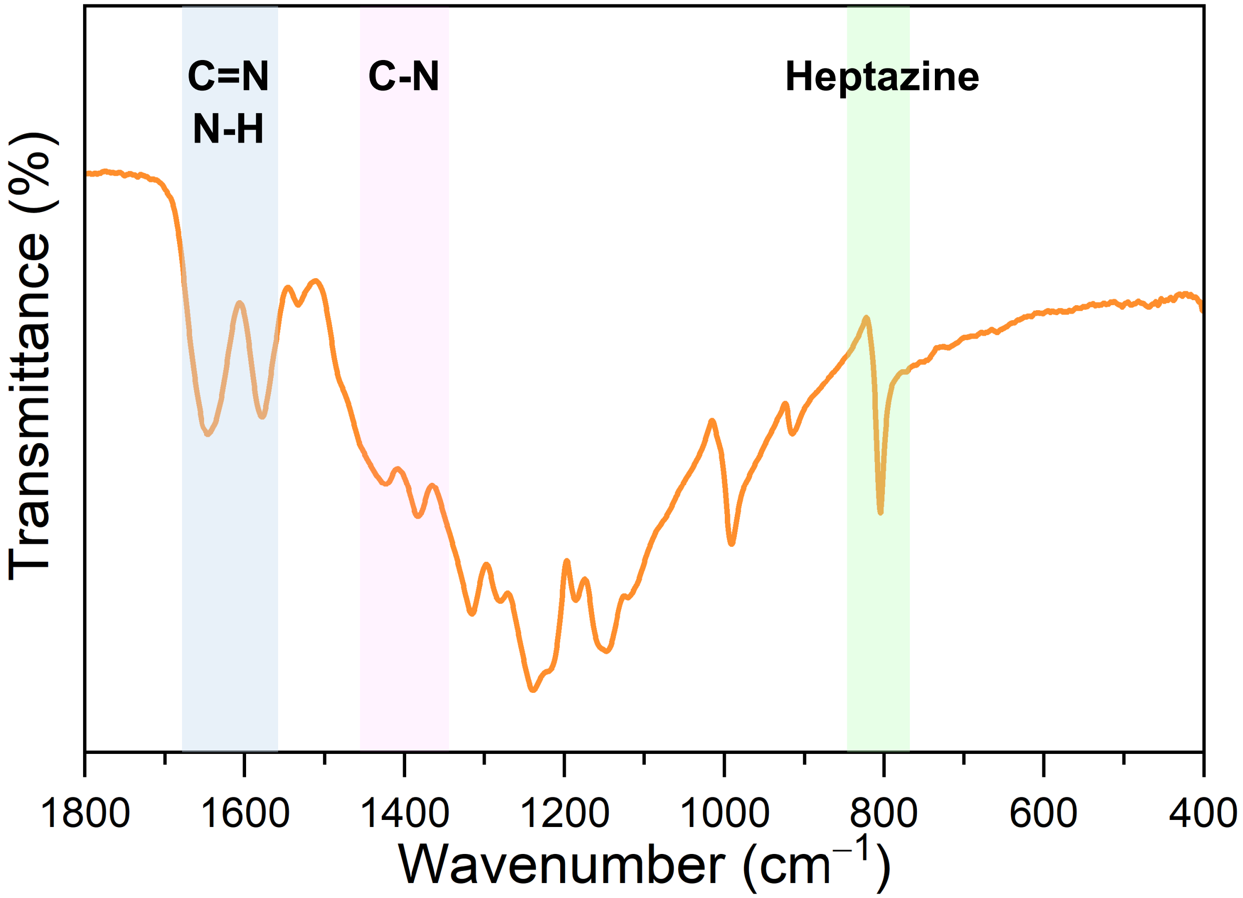

Figure S3: FTIR spectrum of $^{NCN}CN_X$.

S5 Fluorescence Spectroscopy

$^{NCN}CN_X$ shows a broad emission peak at around 450 nm under a excitation wavelength of 370 nm, which can be assigned to the carbon nitride units.[S12,S13] Note that fluorescence peak at 540 nm as a result of surface defects cannot be observed.

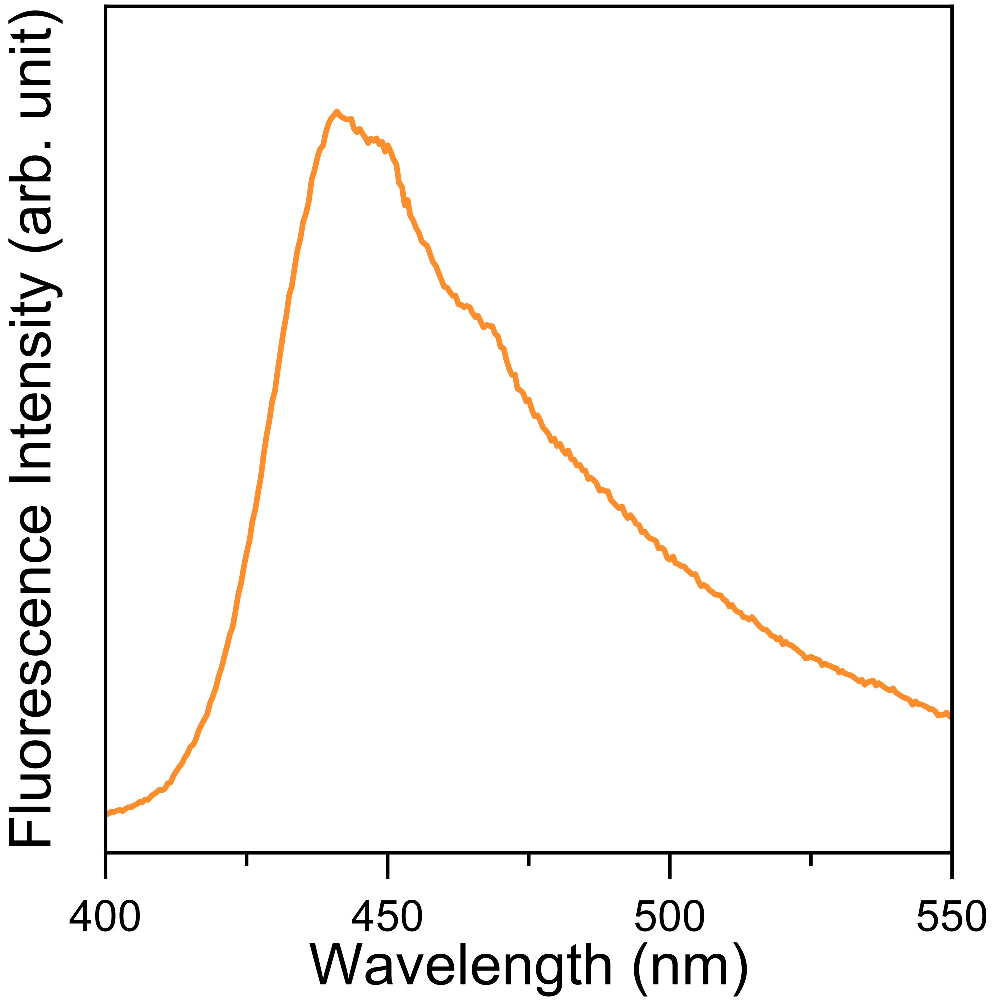

Figure S4: Fluorescence spectrum of $^{NCN}CN_X$ in 0.1 M MOPS, pH 7, 50 mM 4-MBA.

S6 Ultraviolet–Visible (UV−vis) Spectroscopy

$^{NCN}CN_X$ after AM 1.5G illumination shows a newly appeared absorption band at 690 nm, which can be assigned to the blue radicals as previously reported.[S14,S15]

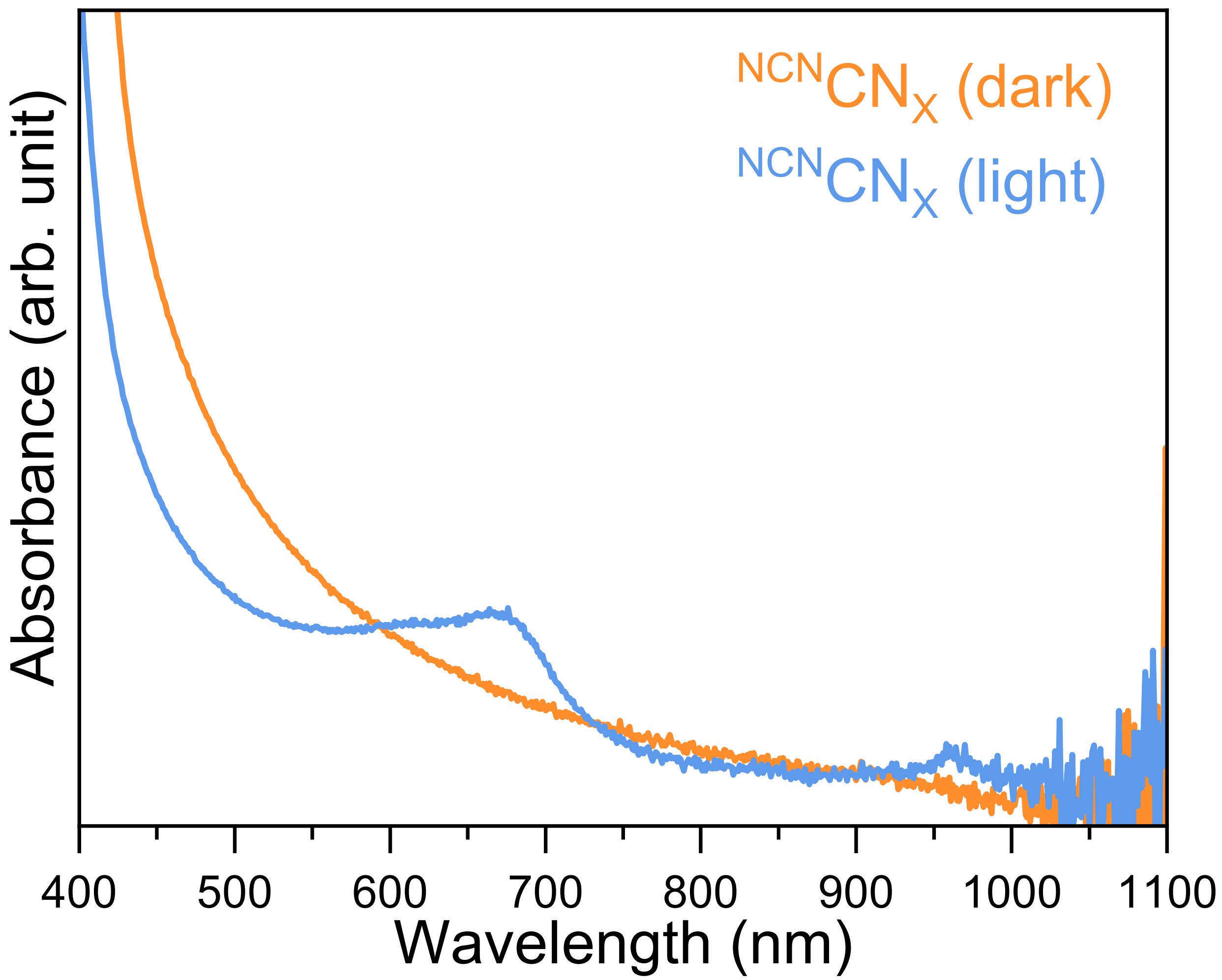

Figure S5: UV−vis spectra of $^{NCN}CN_X$ (light) and $^{NCN}CN_X$ (dark) in 0.1 M MOPS, pH 7, 50 mM 4-MBA.

S7 Zeta Potential Measurements

The blue radicals observed in Figure S5 does not impact the surface charge of $^{NCN}CN_X$ (Figure S6), indicating that these radicals are long-lived and deeply trapped photoelectrons.[S8,S16]

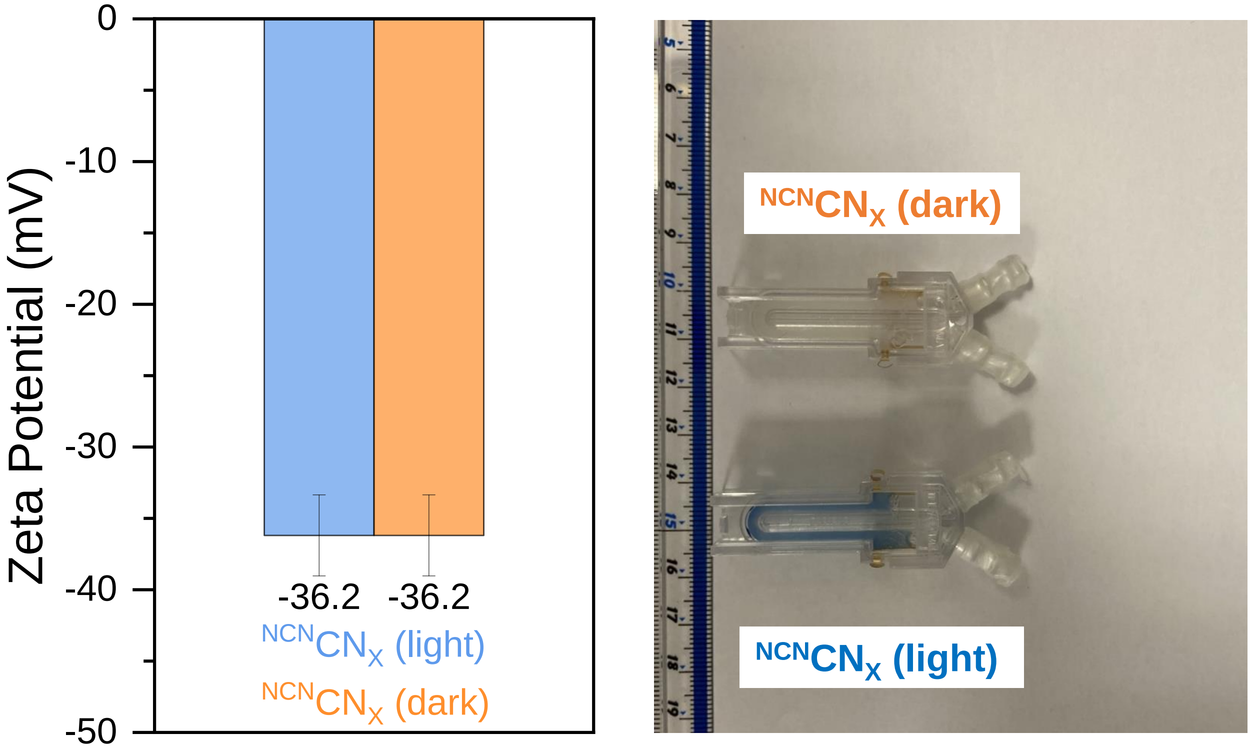

Figure S6: Zeta potential of 2 mg/mL $^{NCN}CN_X$ (light) and $^{NCN}CN_X$ (dark) in 0.1 M MOPS, pH 7, 50 mM 4-MBA.

The solution pH was adjusted by adding 1 M hydrochloric acid (HCl) that was diluted from 37% HCl (Honeywell Fluka).

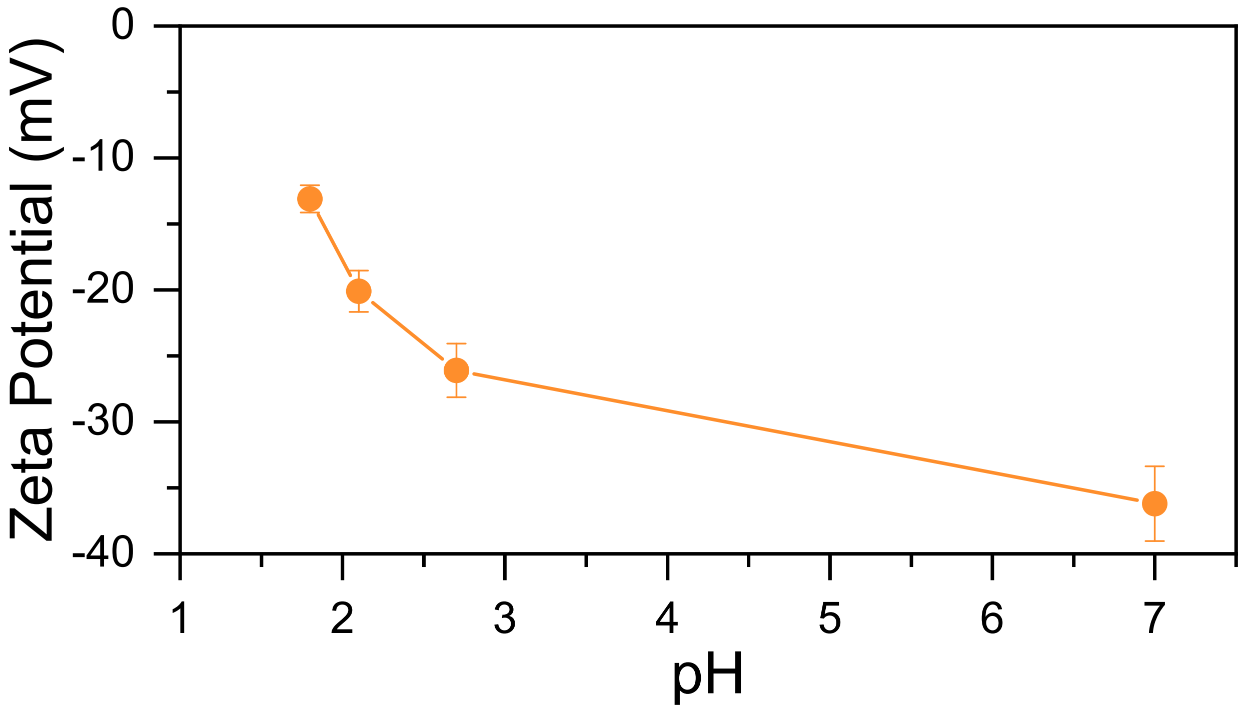

Figure S7: Zeta potential of $^{\mathrm{NCN}}\mathrm{CN_X}$ (2 mg/mL) as a function of pH.

S8 ^{1}H NMR Spectroscopy for Evaluating 4-MBA Oxidation

Detailed peak assignments can be found from Figure S17 to Figure S20.

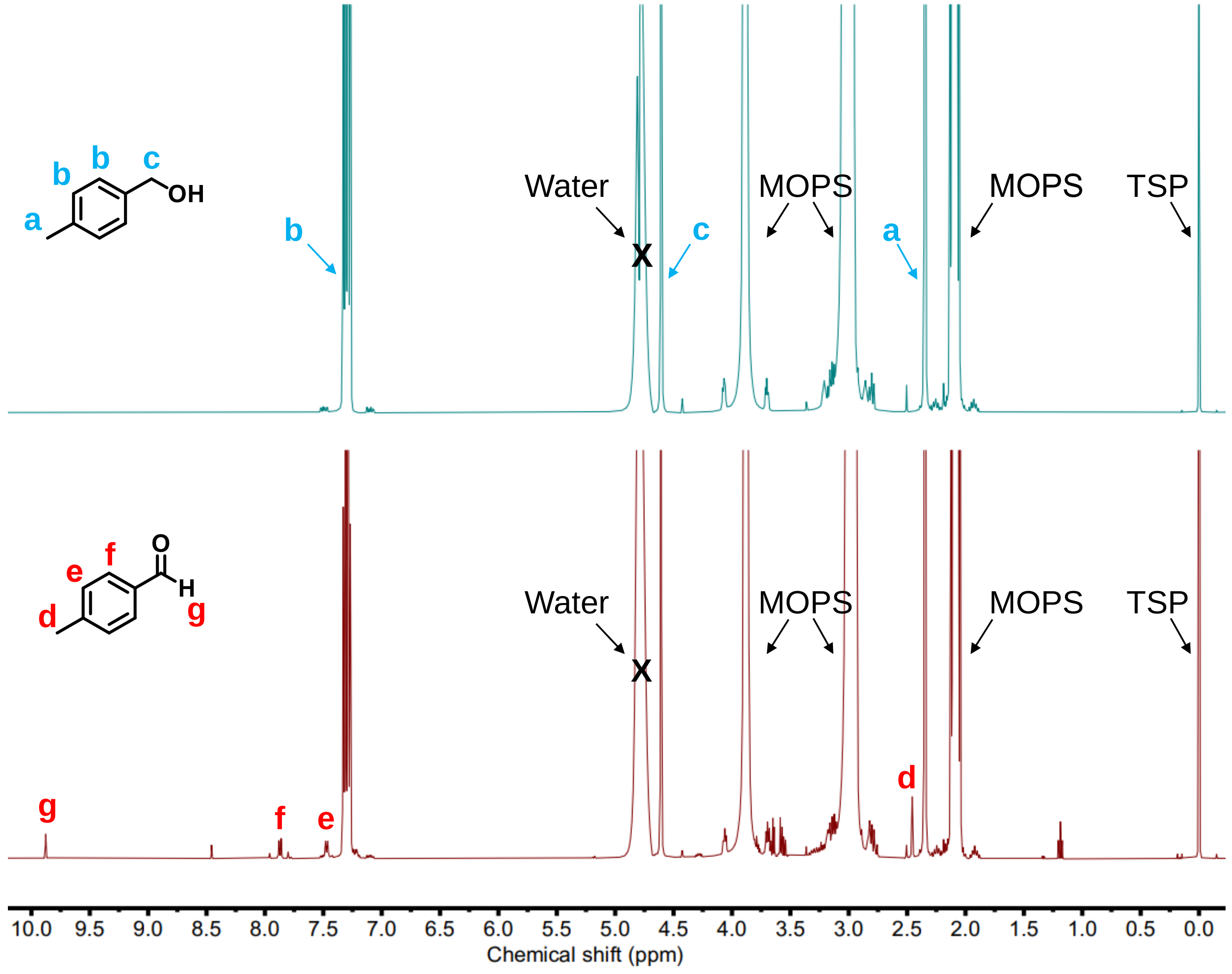

Figure S8: ^{1}H NMR spectra with labelling for 4-methylbenzyl alcohol and 4-methylbenzaldehyde. Top panel: photocatalysis solution at t=0. Bottom panel: photocatalysis solution at t=4h. Condition: 0.1 M MOPS, pH 7, 50 mM 4-MBA, 2 mg/mL $^{NCN}CN_X$, 40 pmol [FeFe]-H_2ase.

S9 High-Performance Liquid Chromatography (HPLC) for 4-MBA Oxidation

HPLC spectrum in Figure S9 shows 4-MBA substrate (retention time 6.1 min) and its oxidation product *p*-tolualdehyde (retention time 8.8 min).

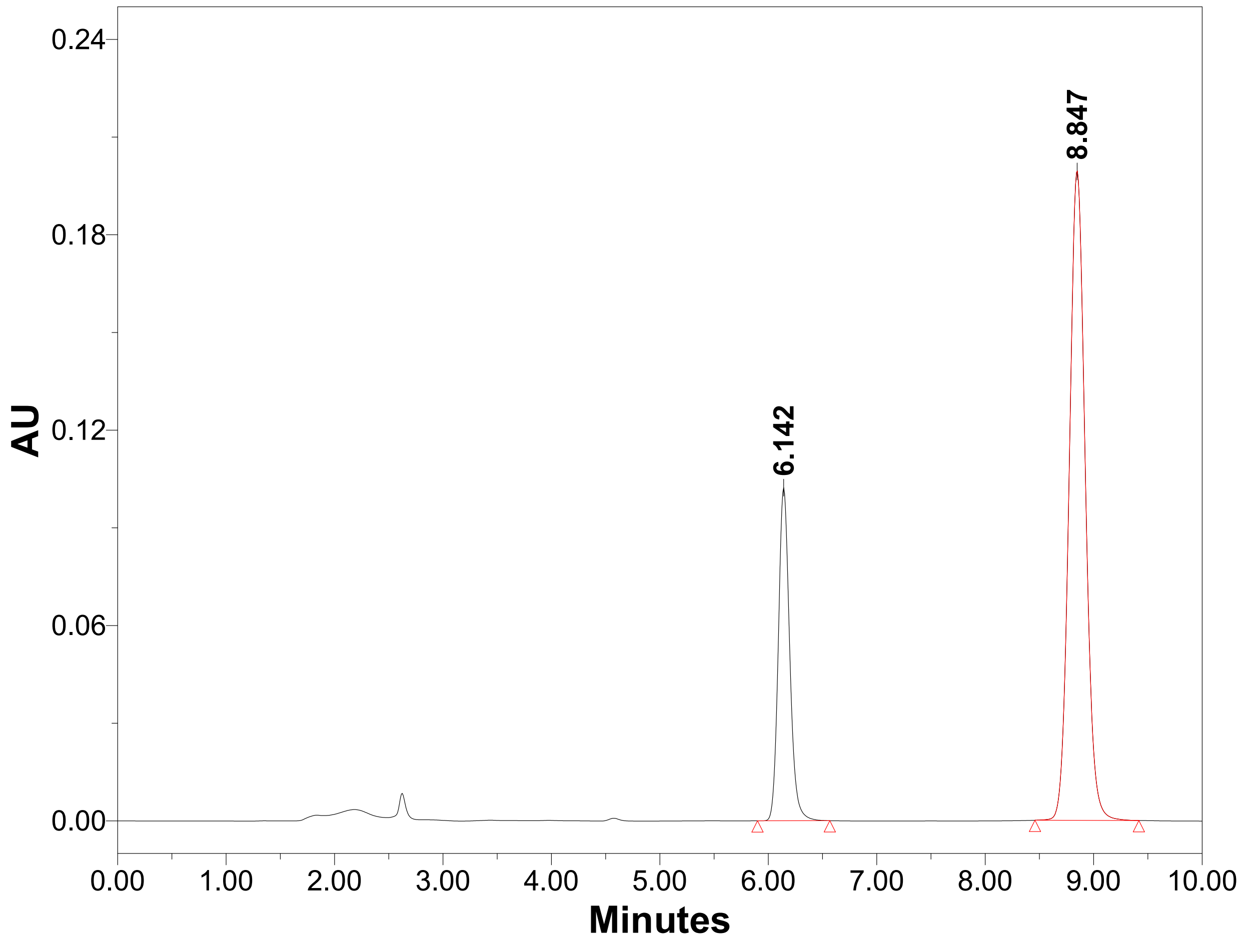

Figure S9: HPLC spectrum of photocatalysis solution at t=24 h with peak assignment for 4-methylbenzyl alcohol (retention time = 6.142 min) and 4-methylbenzaldehyde (retention time = 8.847 min). Condition: 0.1 M MOPS, pH 7, 50 mM 4-MBA, 2 mg/mL $^{NCN}CN_X$, 40 pmol [FeFe]-H_2ase.

HPLC calibration curves in Figure S10 were constructed by linear fitting the integrated HPLC area against known concentrations of stock chemicals.

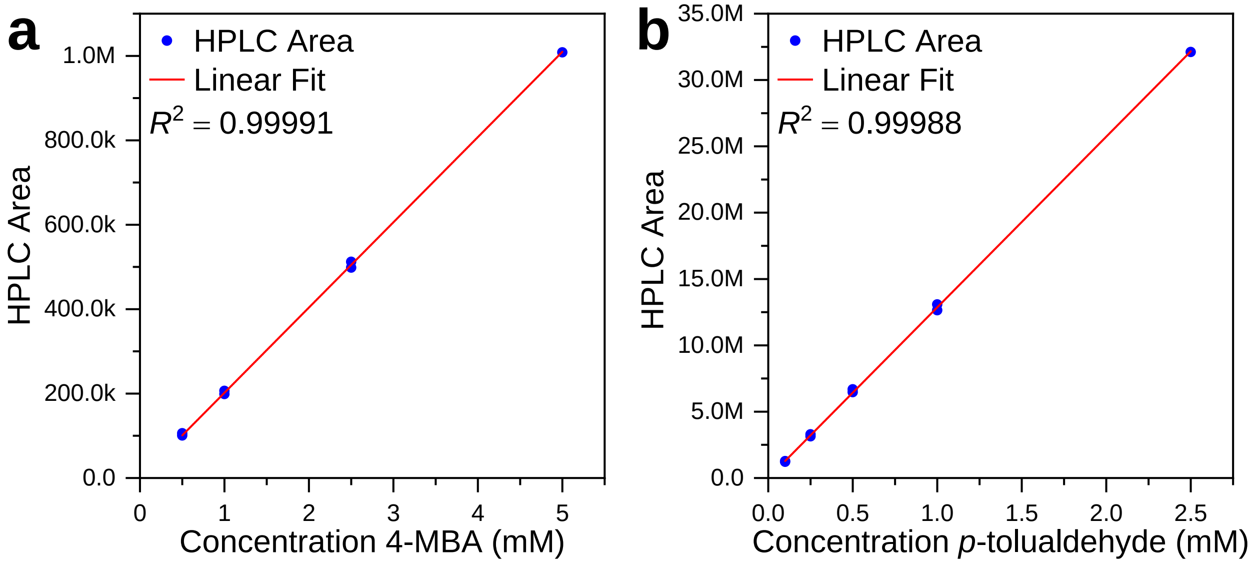

Figure S10: HPLC calibration curves for (a) 4-MBA and (b) *p*-tolualdehyde.

S10 Photograph of Photoreactors Before and After Photocatalysis

As shown in Figure S11, photocatalysis solution turns blue post-reaction (4 hours), indicating the presence of long-lived and deeply trapped photoelectrons as blue radicals.[S8,S15]

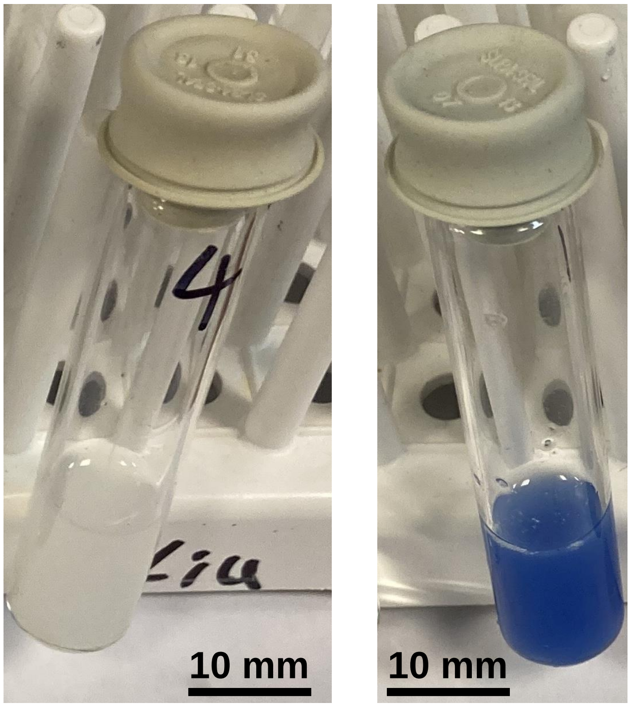

Figure S11: Photograph of as-prepared $^{NCN}CN_X$|[FeFe]+MBA (left panel) and post-reaction (4 hours) $^{NCN}CN_X$|[FeFe]+MBA (right panel). Condition: 0.1 M MOPS, pH 7, 50 mM 4-MBA, 2 mg/mL $^{NCN}CN_X$, 40 pmol [FeFe]-H_2ase.

S11 High-Performance Liquid Chromatography (HPLC) for Glycerol Oxidation

HPLC spectrum in Figure S12 shows glycerol substrate (retention time 20.411 min) and its oxidation products glyceraldehyde (retention time 18.887 min) and dihydroxyacetone (retention time 23.497 min) under refractive index detection.

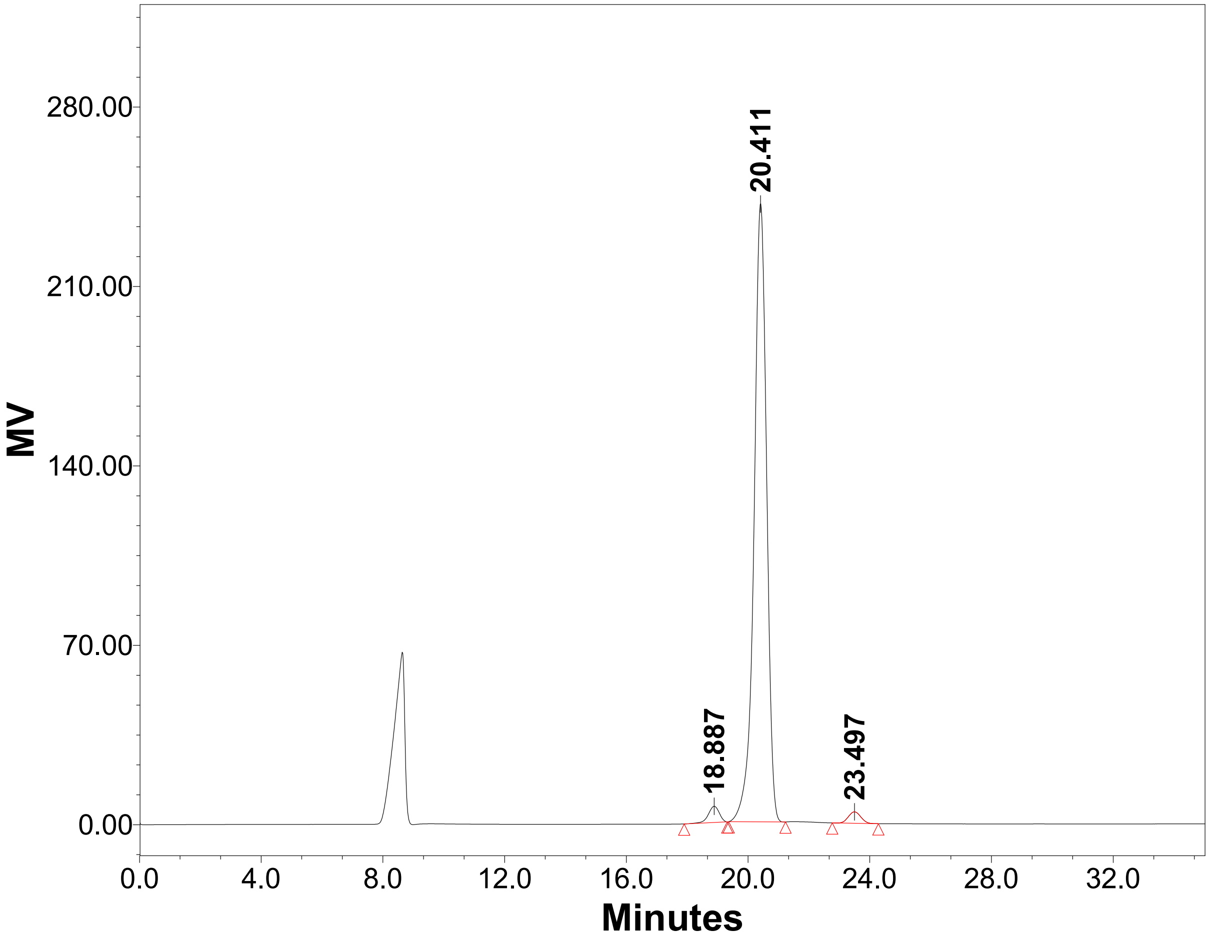

Figure S12: HPLC spectrum of photocatalysis solution at t=2 h with peak assignment for glycerol substrate (retention time 20.411 min) and its oxidation products glyceraldehyde (retention time 18.887 min) and dihydroxyacetone (retention time 23.497 min) under refractive index detection. Condition: pH 7, 50 mM glycerol, 2 mg/mL $^{NCN}CN_X$, 2 wt % Pt.

HPLC-UV (210 nm) spectrum in Figure S13 shows glycerol substrate (retention time around 20 min, at the HPLC detection limit) and its oxidation products glyceraldehyde (retention time 18.523 min) and dihydroxyacetone (retention time 23.125 min).

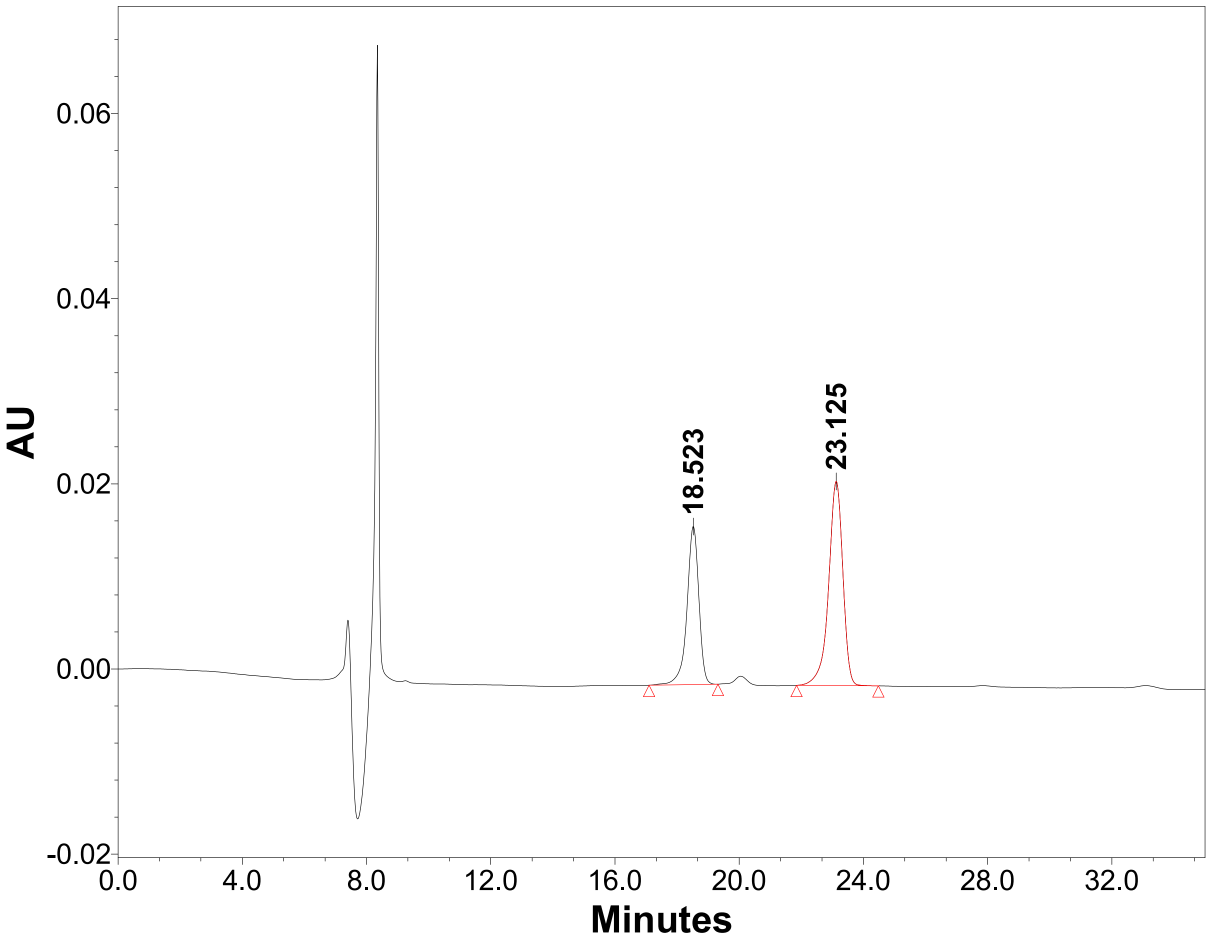

Figure S13: HPLC-UV (210 nm) spectrum of photocatalysis solution at t=2 h with peak assignment for glycerol substrate (retention time around 20 min, at the HPLC detection limit) and its oxidation products glyceraldehyde (retention time 18.523 min) and dihydroxyacetone (retention time 23.125 min). Condition: pH 7, 50 mM glycerol, 2 mg/mL $^{NCN}CN_X$, 2 wt % Pt.

HPLC-UV (270 nm) spectrum in Figure S14 shows glycerol substrate is below the HPLC detection limit and its oxidation products glyceraldehyde (retention time 18.607 min) and dihydroxyacetone (retention time 23.252 min).

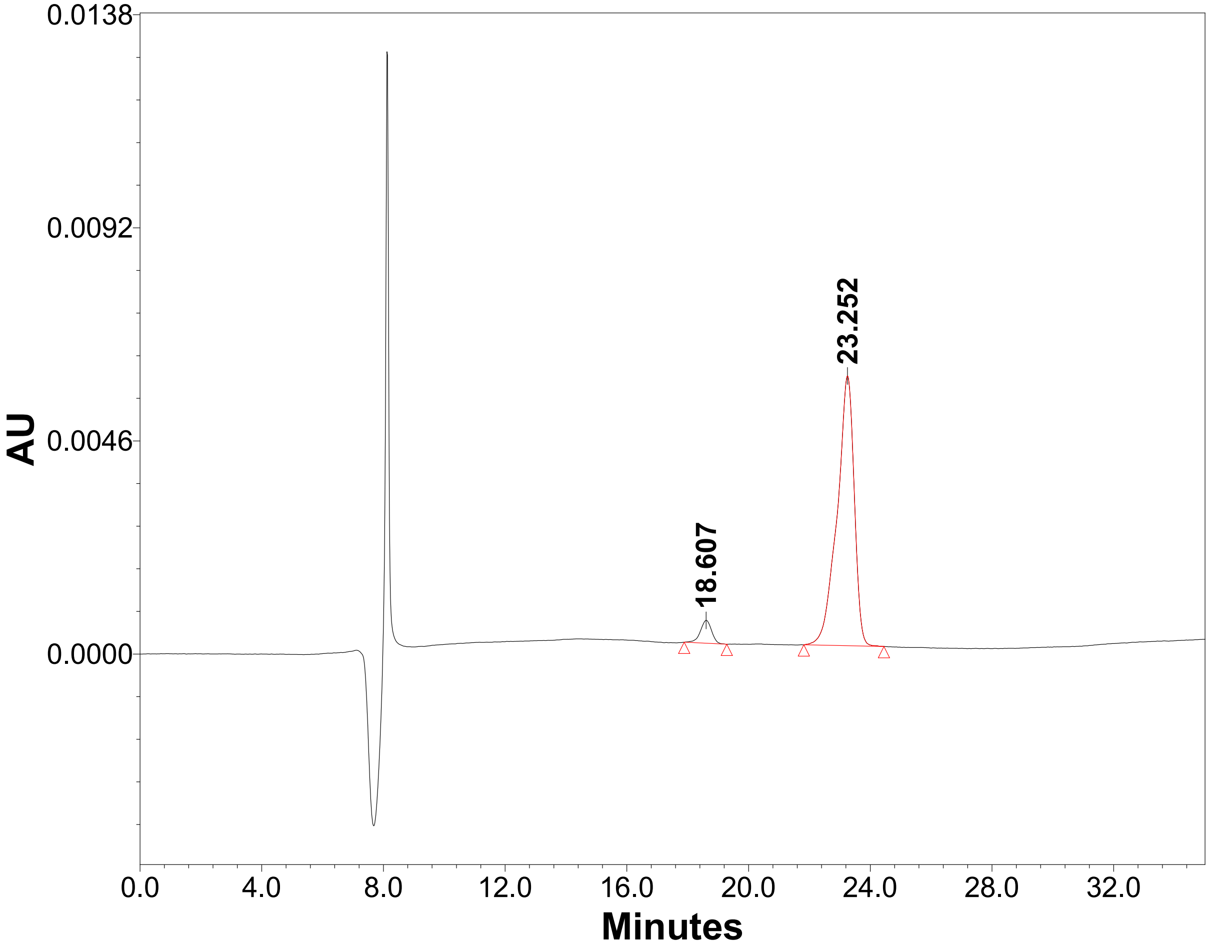

Figure S14: HPLC-UV (270 nm) spectrum of photocatalysis solution at t=2 h with peak assignment for glycerol substrate is below the HPLC detection limit and its oxidation products glyceraldehyde (retention time 18.607 min) and dihydroxyacetone (retention time 23.252 min). Condition: pH 7, 50 mM glycerol, 2 mg/mL $^{NCN}CN_X$, 2 wt % Pt.

HPLC calibration curves in Figure S15 were constructed by linear fitting the integrated HPLC area under UV (210 nm) and UV (270 nm) against known concentrations of stock chemicals of glyceraldehyde and dihydroxyacetone.

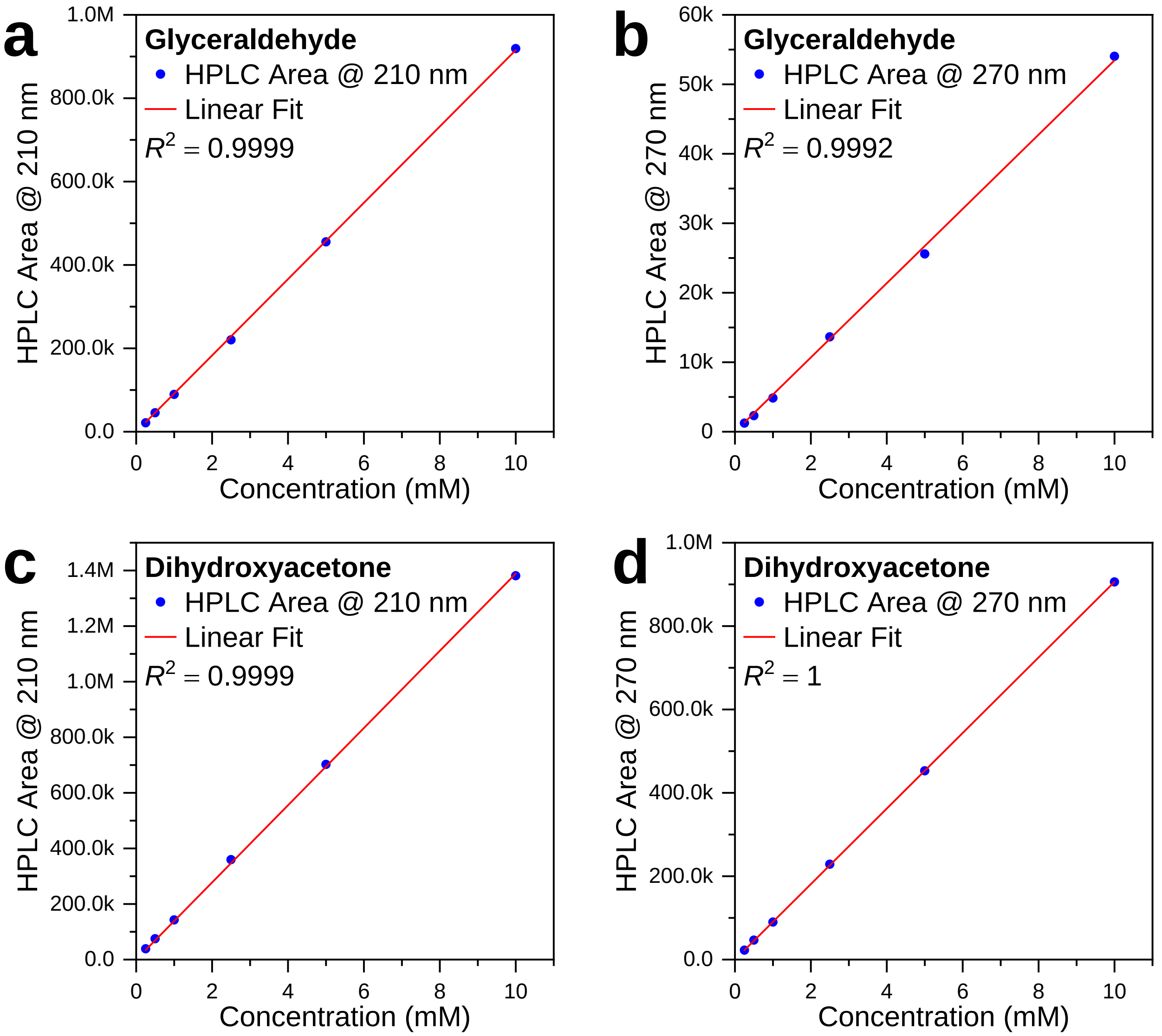

Figure S15: HPLC calibration curves for glyceraldehyde at (a) 210 nm and (b) 270 nm, and for dihydroxyacetone at (c) 210 nm and (d) 270 nm.

S12 Table of Photocatalytic H_2 Evolution under Different Conditions

Table S1: Table of photocatalytic H_2 evolution using $^{NCN}CN_X$ with [FeFe]-H_2ase and [NiFeSe]-H_2ase under different conditions. General conditions: 1 mL anaerobic buffer containing 2 mg $^{NCN}CN_X$, 0.1 M MOPS, 50 mM 4-MBA, pH 7, 40 pmol (unless stated otherwise) H_2ase (either [FeFe] or [NiFeSe]), AM 1.5G irradiation, 100 mW/cm^2, xenon arc lamp, 600 rpm stirring, 25 °C. For mediated electron transfer experiments, 2 mM MV^{2+} was used. Error bars represent the standard deviation for a sample size of 3.

Conditions	H_2 4 h (nmol)	H_2 24 h (nmol)	TON 4 h	TON 24 h	TOF 4 h (h^{-1})	TOF 24 h (h^{-1})
4-MBA+[FeFe]-H_2ase	7 ± 0.4	14 ± 2	175 ± 10	350 ± 50	44 ± 3	15 ± 2
4-MBA+[NiFeSe]-H_2ase	2.4 ± 0.04	7.7 ± 0.21	60 ± 1	192.5 ± 5.25	15 ± 0.25	8 ± 0.22
$^{NCN}CN_X$+4MBA[a]	66 ± 9	164 ± 17				
$^{NCN}CN_X$|[FeFe]-H_2ase	285 ± 24	446 ± 34	7,125 ± 600	11,150 ± 850	1,781 ± 150	465 ± 25
$^{NCN}CN_X$|[NiFeSe]-H_2ase	3.7 ± 0.01	5.1 ± 0.23	92.5 ± 0.25	127.5 ± 5.75	23.1 ± 0.06	5.3 ± 0.24
$^{NCN}CN_X$|[FeFe]-H_2ase+4-MBA	2,987 ± 298	7,925 ± 608	74,675 ± 7,450	198,125 ± 15,200	18,669 ± 1,863	8,255 ± 633
$^{NCN}CN_X$|[NiFeSe]-H_2ase+4-MBA	9 ± 0.3	16 ± 0.8	225 ± 8	400 ± 20	56 ± 2	17 ± 0.8
$^{NCN}CN_X$|[FeFe]-H_2ase+4-MBA+MV	12,123 ± 384	24,014 ± 142	303,075 ± 9,600	600,350 ± 3,550	75,769 ± 2,400	25,015 ± 148
$^{NCN}CN_X$|[NiFeSe]-H_2ase+4-MBA+MV	13,374 ± 845	25,553 ± 1,290	334,350 ± 21,125	638,825 ± 32,250	83,588 ± 5,281	26,618 ± 1,344
$^{NCN}CN_X$|[FeFe]-H_2ase+4-MBA+MV[b]	14,364 ± 667	27,001 ± 978	359,100 ± 16,675	675,025 ± 24,450	89,775 ± 4,169	28,126 ± 1,019
$^{NCN}CN_X$|[NiFeSe]-H_2ase+4-MBA+MV[c]	13,981 ± 431	26,596 ± 730	349,525 ± 10,775	664,900 ± 18,250	87,381 ± 2,694	27,704 ± 760
$^{NCN}CN_X$|[FeFe]-H_2ase+EDTA[d]	3,469 ± 160	10,137 ± 1,446	86,725 ± 4,000	253,425 ± 36,150	21,681 ± 1,000	10,559 ± 1,506

[a] When H_2ase is not presented, there are no physical meanings in TON and TOF in this case.

[b] 80 pmol [FeFe]-H_2ase was added.

[c] 80 pmol [NiFeSe]-H_2ase was added.

[d] 100 mM EDTA was used.

S13 Exclusion Control Experiments for Photocatalytic H_2 Evolution

Figure S16 shows all exclusion control experiments. Trace amount of H_2 is produced with $^{NCN}CN_X$|[FeFe]-H_2ase because of the minor MOPS oxidation as an electron donating reaction. In contrast, no H_2 is detected with $^{NCN}CN_X$|[NiFeSe]-H_2ase, which agrees with electrostatic repulsion. Both [FeFe]-H_2ase and [NiFeSe]-H_2ase are not photoactive and therefore no H_2 is detected in the absence of $^{NCN}CN_X$. $^{NCN}CN_X$ + MBA produced less than 70 nmol of H_2 in 4 hours, indicating $^{NCN}CN_X$ has no intrinsic catalytic activity for H_2 evolution reaction. Note that when no H_2ase is presented in $^{NCN}CN_X$ + MBA, there is no physical meaning in TON for this case.

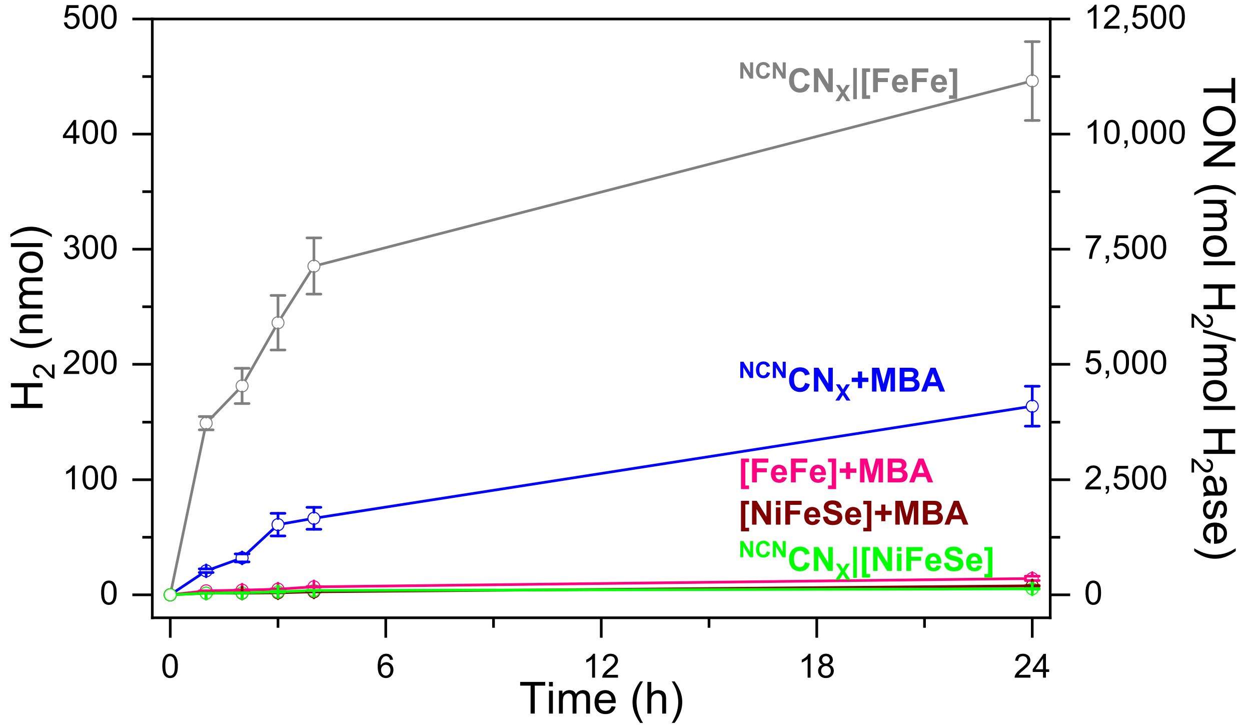

Figure S16: Control experiments for photocatalytic H_2 evolution with $^{NCN}CN_X$ and H_2ases. Conditions: 1 mL anaerobic buffer containing 2 mg $^{NCN}CN_X$, 0.1 M MOPS, 50 mM 4-MBA, pH 7, 40 pmol H_2ase (either *CpI* [FeFe] or *Dv*H [NiFeSe]), AM 1.5G irradiation, 600 rpm stirring, 25 °C. Error bars represent the standard deviation for a sample size of 3.

S14 ^{1}H NMR Spectroscopy for Evaluating MOPS Oxidation

The following references were used to support peak assignment in Figure S17:

4-methylbenzyl alcohol ($CH_3C_6H_4CH_2OH$),[S14,S17]

and MOPS ($C_7H_{15}NO_4S$).[S18]

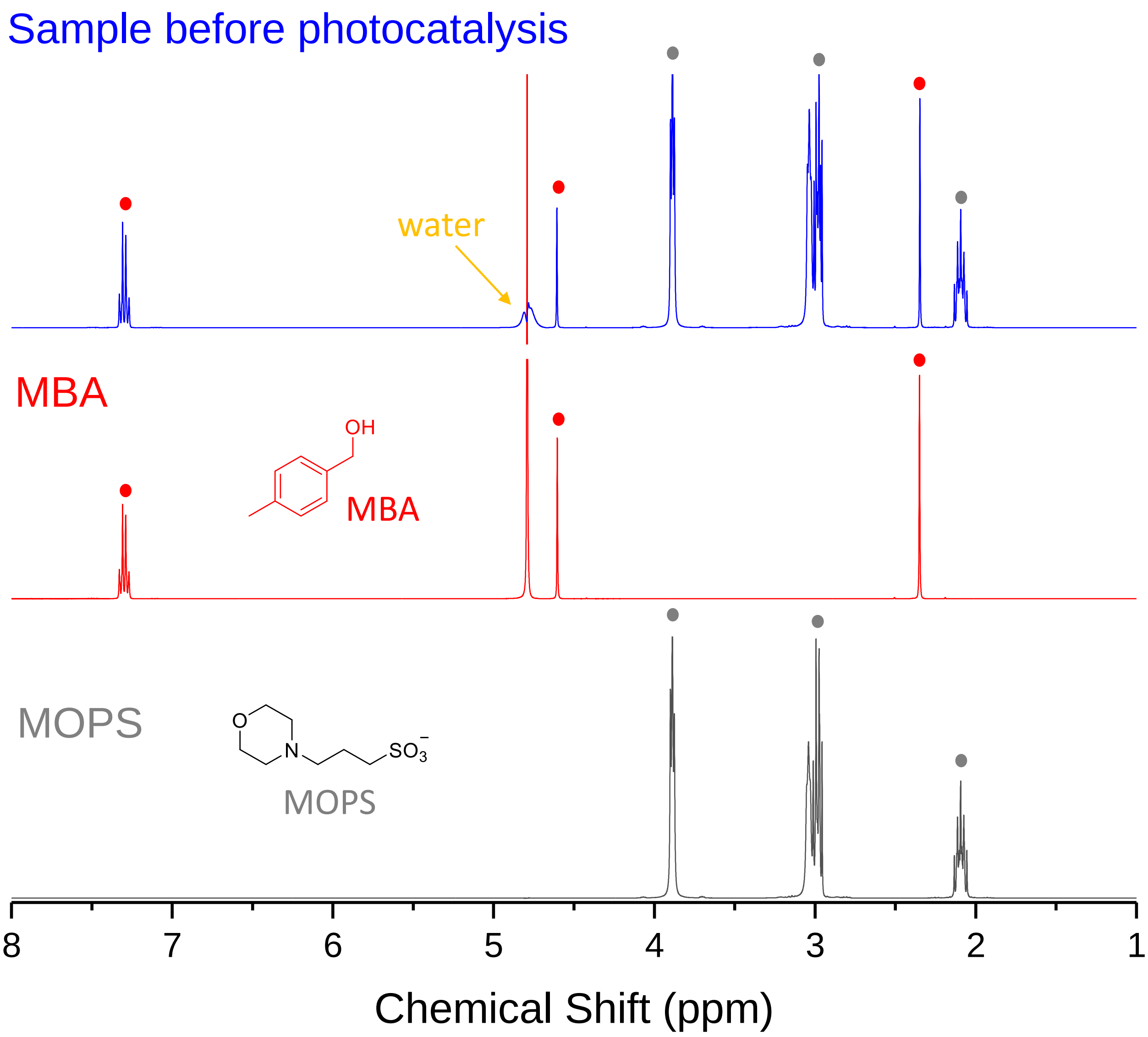

Figure S17: ^{1}H NMR spectra of sample before photocatalysis (blue) with labelling for 4-methylbenzyl alcohol and MOPS. Condition: 0.1 M MOPS, pH 7, 50 mM 4-MBA, 2 mg/mL $^{NCN}CN_X$, 40 pmol [FeFe]-H_2ase.

The following references were used to support peak assignment in Figure S18:

4-methylbenzyl alcohol ($CH_3C_6H_4CH_2OH$),[S14,S17]

4-methylbenzaldehyde ($CH_3C_6H_4CHO$),[S14,S17]

MOPS ($C_7H_{15}NO_4S$),[S18]

and $MOPS_{OX}$ (not refer to a clean product as MOPS oxidation is complex).[S19]

$MOPS_{OX}$ was obtained by electrochemically oxidizing MOPS buffer for 1 hour at a constant current of 3 mA using 2 platinum meshes as working and counter electrodes.

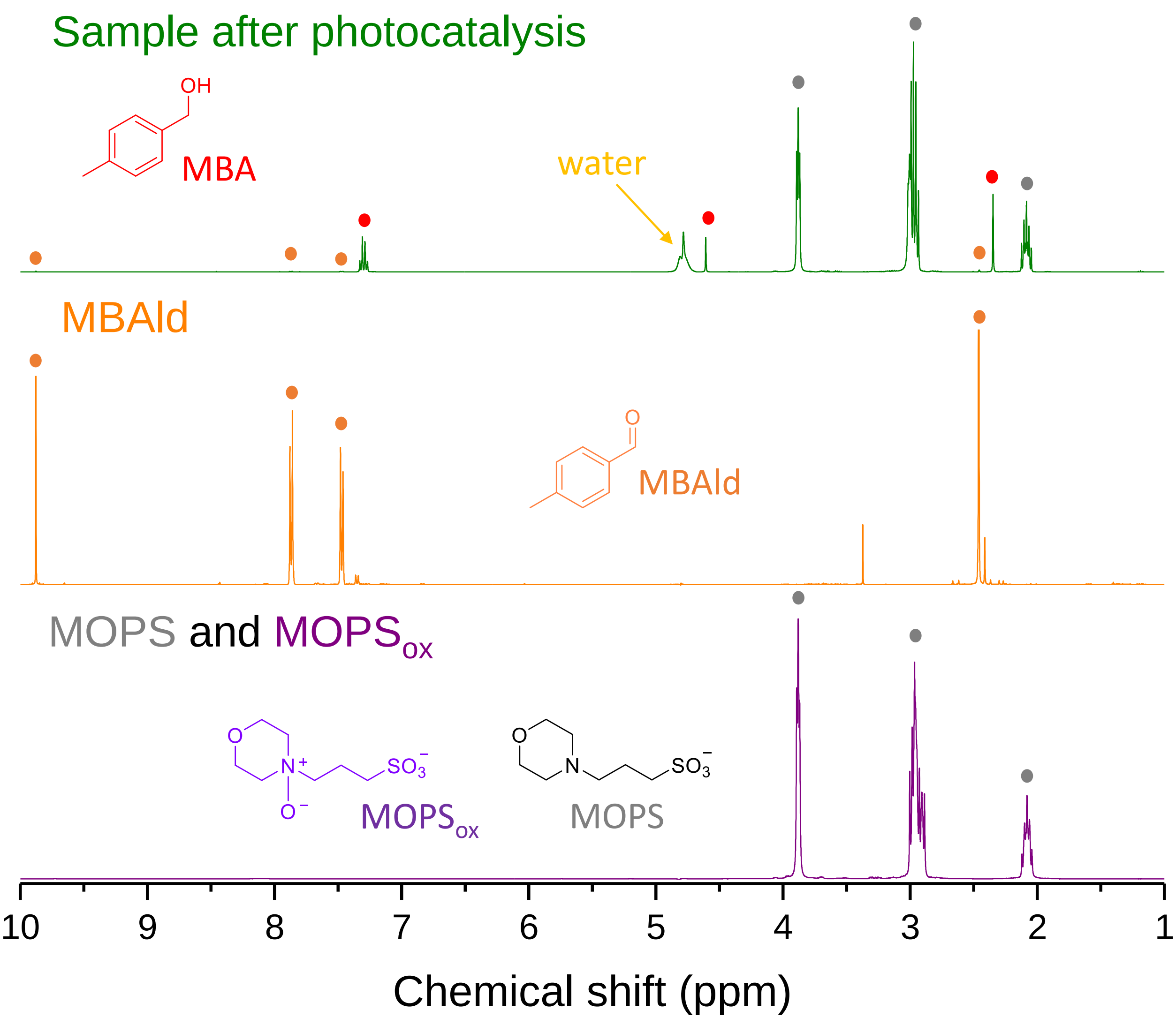

Figure S18: ^{1}H NMR spectra of sample after 4 h photocatalysis (green) with labelling for 4-methylbenzyl alcohol, 4-methylbenzaldehyde, MOPS, and $MOPS_{OX}$. Condition: 0.1 M MOPS, pH 7, 50 mM 4-MBA, 2 mg/mL $^{NCN}CN_X$, 40 pmol [FeFe]-H_2ase.

The following references were used to support peak assignment in Figure S19:

4-methylbenzyl alcohol ($CH_3C_6H_4CH_2OH$),[S14,S17]

4-methylbenzaldehyde ($CH_3C_6H_4CHO$),[S14,S17]

MOPS ($C_7H_{15}NO_4S$),[S18]

and $MOPS_{OX}$ (not refer to a clean product as MOPS oxidation is complex).[S19]

$MOPS_{OX}$ was obtained by electrochemically oxidizing MOPS buffer for 1 hour at a constant current of 3 mA using 2 platinum meshes as working and counter electrodes.

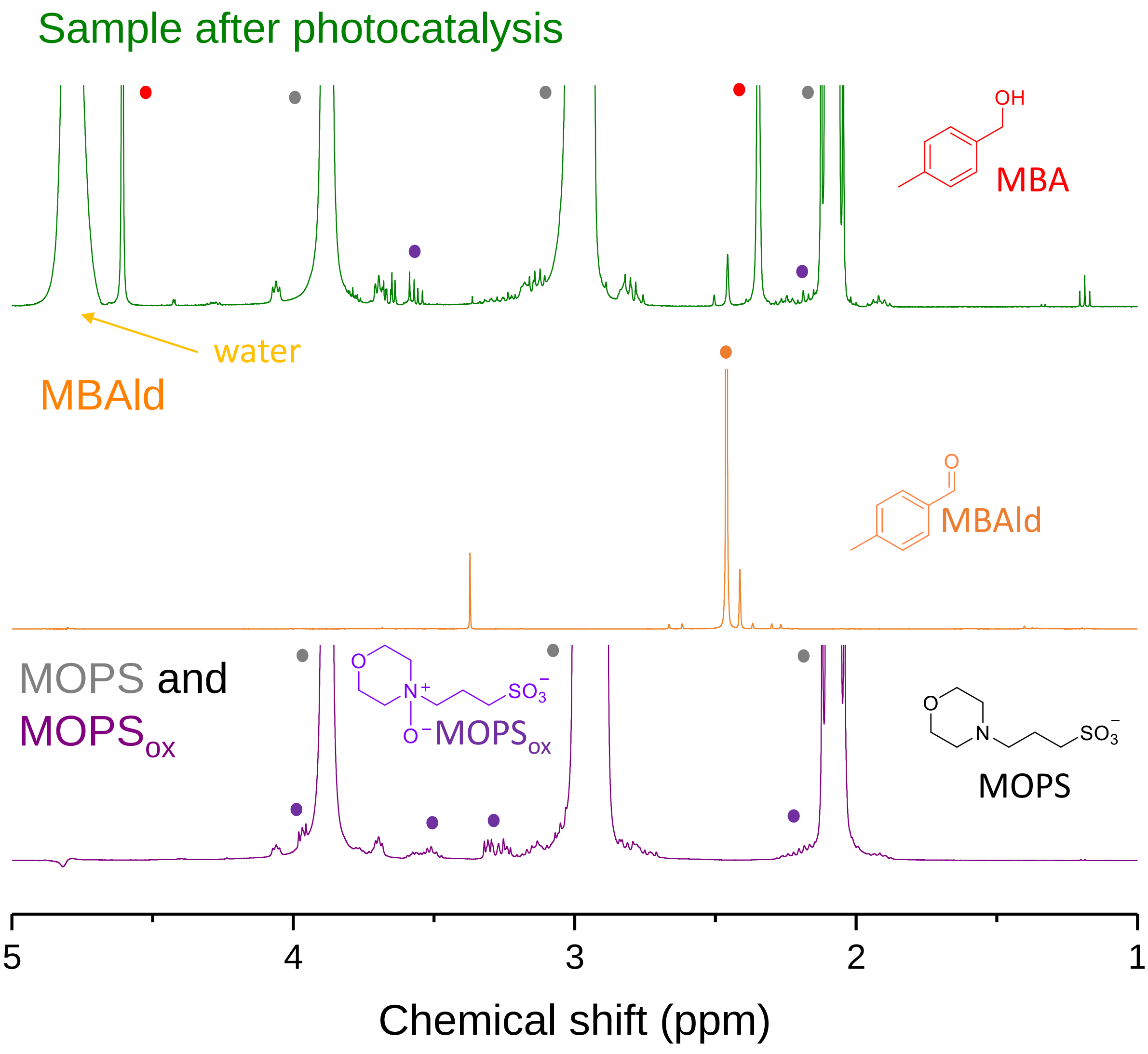

Figure S19: Zoom up of ^{1}H NMR spectra, from 5 to 1 ppm, of sample after 4 h photocatalysis (green) with labelling for 4-methylbenzyl alcohol, 4-methylbenzaldehyde, MOPS, and $MOPS_{OX}$. Condition: 0.1 M MOPS, pH 7, 50 mM 4-MBA, 2 mg/mL $^{NCN}CN_X$, 40 pmol [FeFe]-H_2ase.

The following references were used to support peak assignment in Figure S20:

4-methylbenzyl alcohol ($CH_3C_6H_4CH_2OH$),[S14,S17]

4-methylbenzaldehyde ($CH_3C_6H_4CHO$),[S14,S17]

MOPS ($C_7H_{15}NO_4S$),[S18]

and $MOPS_{OX}$ (not refer to a clean product as MOPS oxidation is complex).[S19]

$MOPS_{OX}$ was obtained by electrochemically oxidizing MOPS buffer for 1 hour at a constant current of 3 mA using 2 platinum meshes as working and counter electrodes.

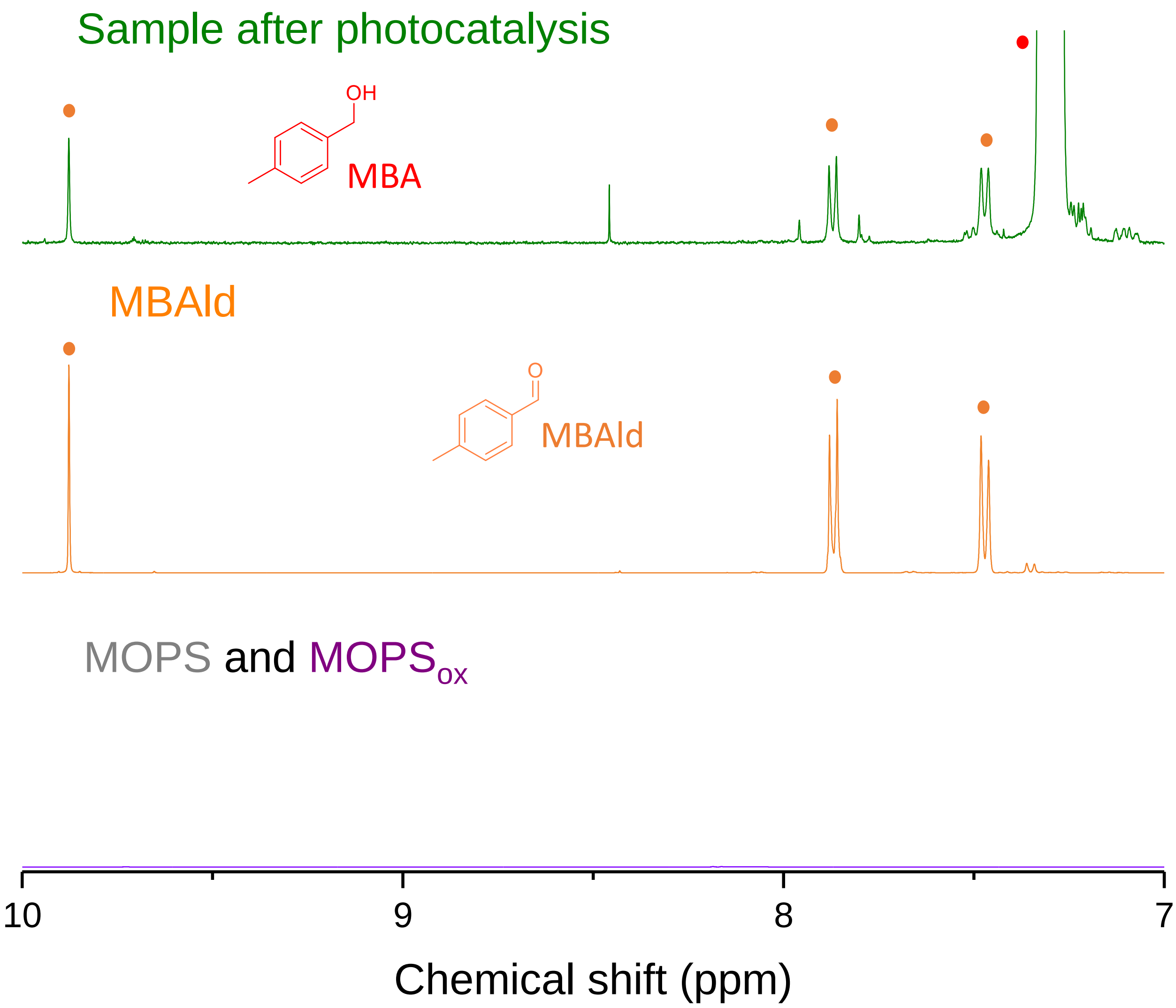

Figure S20: Zoom up of ^1H NMR spectra, from 10 to 7 ppm, of sample after 4 h photocatalysis (green) with labelling for 4-methylbenzyl alcohol, 4-methylbenzaldehyde, MOPS, and $MOPS_{OX}$. Condition: 0.1 M MOPS, pH 7, 50 mM 4-MBA, 2 mg/mL $^{NCN}CN_X$, 40 pmol [FeFe]-H_2ase.

S15 Photocatalytic H_2 Evolution with $^{NCN}CN_X$|[FeFe]-H_2ase Assemblies in MOPS and Phosphate

Upon replacing MOPS buffer with pH 7 phosphate buffer, the reaction is terminated in the absence of 4-MBA, accompanied by a ∼27% decrease in photocatalytic activity in the presence of 4-MBA (Figure S21, Table S2). This observation is in line with literature that MOPS as a standard Good's buffer can maintain high *in vitro* biochemical and biological activities.[S20]

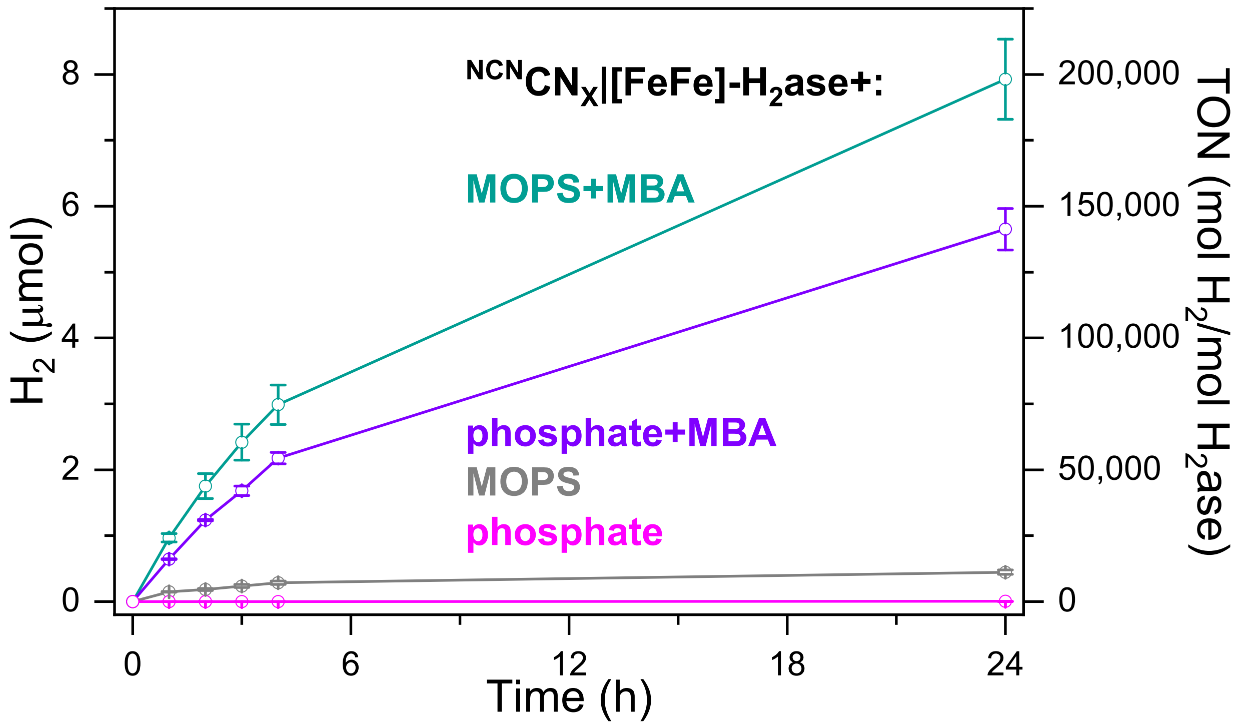

Figure S21: Photocatalytic H_2 evolution with $^{NCN}CN_X$|[FeFe]-H_2ase assemblies in MOPS and phosphate. Conditions: 1 mL anaerobic buffer containing 2 mg $^{NCN}CN_X$, pH 7 buffer (either 0.1 M MOPS or 0.1 M phosphate), 50 mM 4-MBA, 40 pmol *CpI* [FeFe]-H_2ase, AM 1.5G irradiation, 600 rpm stirring, 25 °C. Error bars represent the standard deviation for a sample size of 3.

S16 Table of Photocatalytic H_2 Evolution with $^{NCN}CN_X$|[FeFe]-H_2ase Assemblies in MOPS and Phosphate

Table S2: Photocatalytic H_2 evolution with $^{NCN}CN_X$|[FeFe]-H_2ase assemblies in MOPS and phosphate. Conditions: 1 mL anaerobic buffer containing 2 mg $^{NCN}CN_X$, pH 7 buffer (either 0.1 M MOPS or 0.1 M phosphate), 50 mM 4-MBA, 40 pmol *CpI* [FeFe]-H_2ase, AM 1.5G irradiation, 100 mW/cm^2, xenon arc lamp, 600 rpm stirring, 25 °C. Error bars represent the standard deviation for a sample size of 3.

Conditions	H_2 4 h (nmol)	H_2 24 h (nmol)	TON 4 h	TON 24 h	TOF 4 h (h^{-1})	TOF 24 h (h^{-1})
$^{NCN}CN_X$|[FeFe]-H_2ase+MOPS	285 ± 24	446 ± 34	7,125 ± 600	11,150 ± 850	1,781 ± 150	465 ± 25
$^{NCN}CN_X$|[FeFe]-H_2ase+phosphate	1.3 ± 0.004	2.1 ± 0.2	32.5 ± 0.1	52.5 ± 5	8.1 ± 0.025	2.2 ± 0.21
$^{NCN}CN_X$|[FeFe]-H_2ase+MOPS+4-MBA	2,987 ± 298	7,925 ± 608	74,675 ± 7,450	198,125 ± 15,200	18,669 ± 1,863	8,255 ± 633
$^{NCN}CN_X$|[FeFe]-H_2ase+phosphate+4-MBA	2,176 ± 86	5,650 ± 313	54,440 ± 2,150	141,250 ± 7825	13,610 ± 537.5	5,885 ± 326

S17 Ratio of DET to MET

The efficiency of DET is qualitatively determined by the DET:MET ratio, defined by the ratio of H_2 yield in the absence of MV (DET) and in the presence of MV (MET) as follows:

$$\frac{\text{DET}}{\text{MET}} = \frac{\text{H}_2 \text{ yield without MV}}{\text{H}_2 \text{ yield with MV}}$$

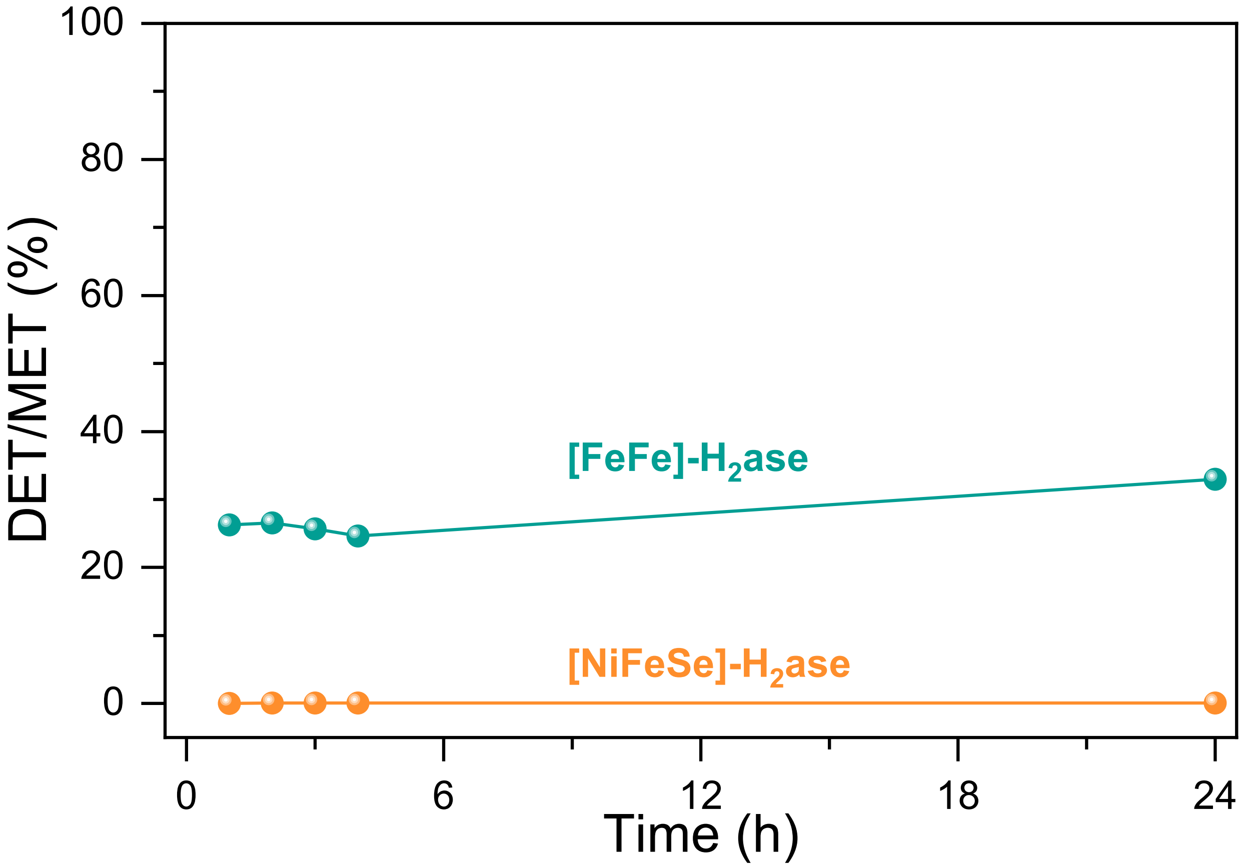

Figure S22: Ratio of DET to MET, determined by H_2 yield without (DET) and with (MET) MV. DET conditions: 1 mL anaerobic buffer containing 2 mg $^{NCN}CN_X$, 0.1 M MOPS, 50 mM 4-MBA, pH 7, 40 pmol H_2ase (either *CpI* [FeFe] or *Dv*H [NiFeSe]), AM 1.5G irradiation, 600 rpm stirring, 25 °C. MET conditions: 1 mL anaerobic buffer containing 2 mg $^{NCN}CN_X$, 0.1 M MOPS, 50 mM 4-MBA, pH 7, 2 mM MV^{2+}, 40 pmol H_2ase (either *CpI* [FeFe] or *Dv*H [NiFeSe]), AM 1.5G irradiation, 600 rpm stirring, 25 °C.

S18 Comparison among State-of-the-art Carbon Nitride Photocatalysts and [FeFe]-H_2ase Assemblies for H_2 Evolution

Table S3: Comparison among state-of-the-art carbon nitride photocatalysts[S21,S22] and [FeFe]-H_2ase assemblies[S23,S24] for H_2 evolution.

Conditions	H_2 4 h (μmol)	TON 4 h	TOF 4 h (h^{-1})	H_2 4 h (μmol h^{-1} g^{-1})	AQE	Ref.
$^{NCN}CN_X$|[FeFe]-H_2ase+4-MBA[a]	3.0 ± 0.3	74,675 ± 7,450	18,669 ± 1,863	375 ± 37.5	0.35% @ 450 nm	This work
$^{NCN}CN_X$|[FeFe]-H_2ase+4-MBA+MV[a]	12.1 ± 0.4	303,075 ± 9,600	75,769 ± 2,400	1,512.5 ± 50	1.4% @ 450 nm	This work
g-C_3N_4|Pt+TEOA[b]	340.4	220	55	8,510	5.1% @ 420 nm	[S21]
g-C_3N_4|Pt+TEOA[c]	319.2	N.A.	N.A.	1,596	3.56% @ 420 nm	[S22]
CdTe|[FeFe]-H_2ase+AsA[d] (5 min)	N.A.	7,500	25 s^{-1}	N.A.	1.8% @ AM 1.5	[S23]
CDs|[FeFe]-H_2ase+TEOA[e]	1.5	6,000	1,500	N.A.	1.7% @ 420 nm	[S24]
CDs|[FeFe]-H_2ase+TEOA+MV[e]	2	8,000	2,000	N.A.	N.A.	[S24]

[a] Illumination conditions: AM 1.5G, 100 mW/cm^2, xenon arc lamp.

[b] Illumination conditions: >420 nm, Xe lamp.

[c] Illumination conditions: >400 nm, Xe lamp.

[d] In this case, H_2 yield, TON, and TOF are all determined by a 5 min photocatalysis experiment, different from the 4 h experiment stated in the header row. AsA stands for ascorbic acid. Illumination conditions: ∼AM 3, ∼70 mW/cm^2, halogen projector lamp.

[e] Illumination conditions: 50 mW/cm^2, LED lamp.

S19 Nyquist Plots of Impedance Response at Different Potentials

Figure S23 shows the voltage-dependent impedance response, where a more negative applied potential yields a diminished semicircular feature in the Nyquist plots, indicating reduced charge transfer resistance. This observation is a result of the introduction of a larger band bending, resulting in improved separation of photogenerated charges.[S25,S26] Consequently, a greater population of free charge carriers is localized within the semiconductor, increasing the conductivity of $^{NCN}CN_X$.

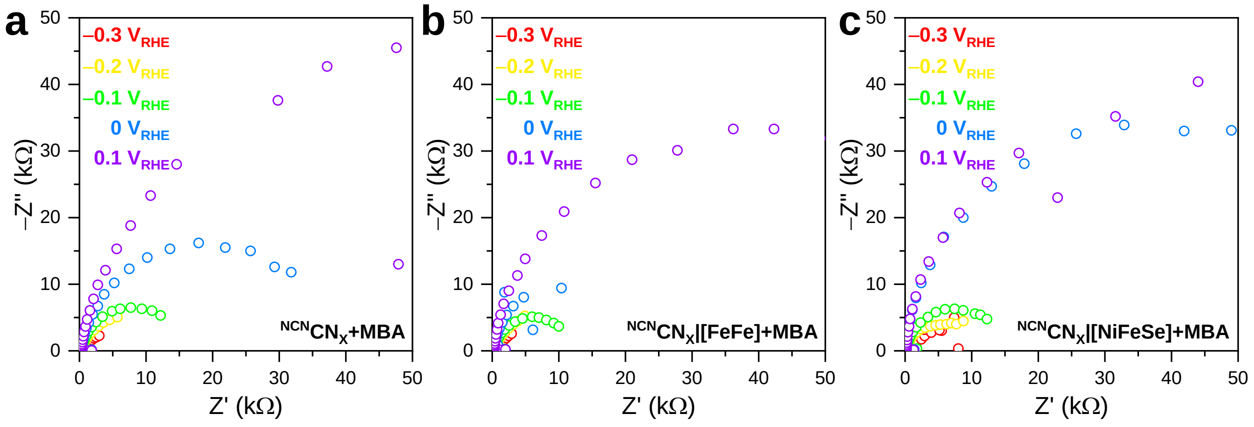

Figure S23: Nyquist plots of impedance response at 5 different potentials for (a) $^{NCN}CN_X$, (b) $^{NCN}CN_X$ with [FeFe]-H_2ase, and (c) $^{NCN}CN_X$ with [NiFeSe]-H_2ase. Conditions: 20 mL anaerobic buffer containing 0.1 M MOPS, 50 mM 4-MBA, pH 7, AM 1.5G irradiation, 25 °C.

S20 High Frequency Impedance Response

The high frequency (10 kHz to 1 MHz) RC response in the Nyquist plots (Figure S24a) and in the Bode phase plots (Figure S24b) represents the impedance response of the conductive substrate.

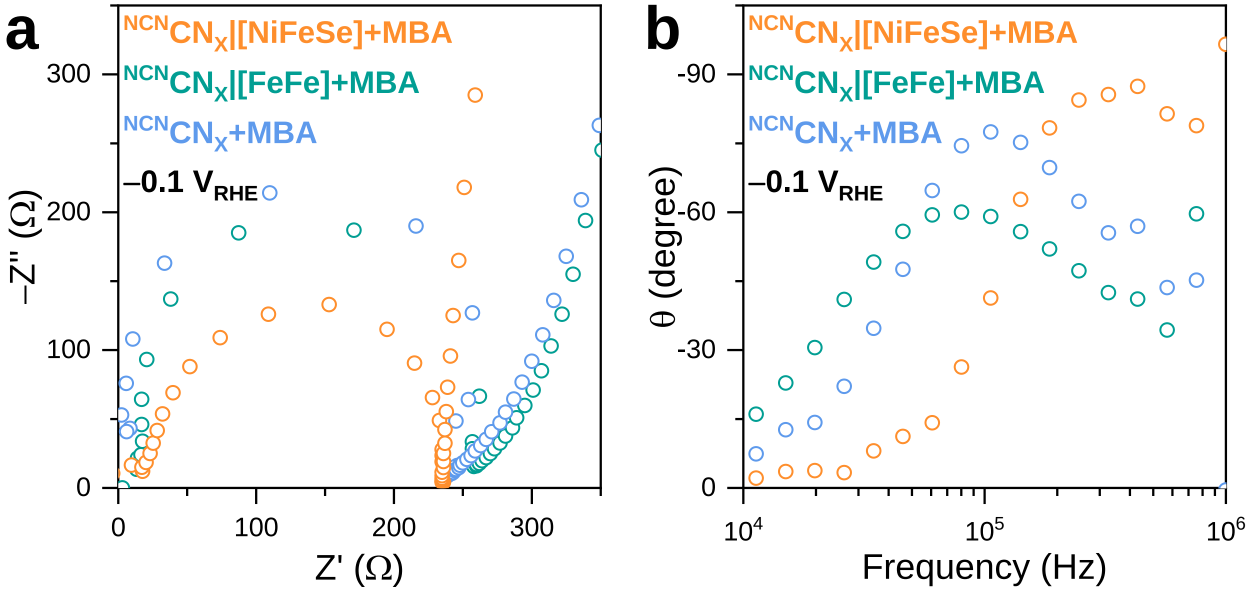

Figure S24: (a) Nyquist plots and (b) Bode phase plots of impedance response at high frequency region. Conditions: 20 mL anaerobic buffer containing 0.1 M MOPS, 50 mM 4-MBA, pH 7, AM 1.5G irradiation, 25 °C.

References

(S1) Liu, Y.; Webb, S.; Moreno-Garcia, P.; Kulkarni, A.; Maroni, P.; Broekmann, P.; Milton, R. D. Facile Functionalization of Carbon Electrodes for Efficient Electroenzymatic Hydrogen Production. *JACS Au* **2023**, *3*, 124–130.

(S2) Marques, M. C.; Tapia, C.; Gutiérrez-Sanz, O.; Ramos, A. R.; Keller, K. L.; Wall, J. D.; De Lacey, A. L.; Matias, P. M.; Pereira, I. A. The direct role of selenocysteine in [NiFeSe] hydrogenase maturation and catalysis. *Nat. Chem. Biol.* **2017**, *13*, 544–550.

(S3) Liu, J.; Liu, Y.; Liu, N.; Han, Y.; Zhang, X.; Huang, H.; Lifshitz, Y.; Lee, S.-T.; Zhong, J.; Kang, Z. Metal-free efficient photocatalyst for stable visible water splitting via a two-electron pathway. *Science* **2015**, *347*, 970–974.

(S4) Lau, V. W.-h.; Moudrakovski, I.; Botari, T.; Weinberger, S.; Mesch, M. B.; Duppel, V.; Senker, J.; Blum, V.; Lotsch, B. V. Rational design of carbon nitride photocatalysts by identification of cyanamide defects as catalytically relevant sites. *Nat. Commun.* **2016**, *7*, 12165.

(S5) Uekert, T.; Kasap, H.; Reisner, E. Photoreforming of nonrecyclable plastic waste over a carbon nitride/nickel phosphide catalyst. *J. Am. Chem. Soc.* **2019**, *141*, 15201–15210.

(S6) Li, R.; Li, C. *Advances in Catalysis: Chapter One - Photocatalytic Water Splitting on Semiconductor-Based Photocatalysts*; Elsevier, 2017; Vol. 60; pp 1–57.

(S7) Sauerbrey, G. Verwendung von Schwingquarzen zur Wägung dünner Schichten und zur Mikrowägung. *Zeitschrift für physik* **1959**, *155*, 206–222.

(S8) Pulignani, C.; Mesa, C. A.; Hillman, S. A.; Uekert, T.; Giménez, S.; Durrant, J. R.; Reisner, E. Rational Design of Carbon Nitride Photoelectrodes with High Activity Toward Organic Oxidations. *Angew. Chem. Int. Ed.* **2022**, *61*, e202211587.

(S9) Peters, J. W.; Lanzilotta, W. N.; Lemon, B. J.; Seefeldt, L. C. X-ray crystal structure of the Fe-only hydrogenase (CpI) from *Clostridium pasteurianum* to 1.8 angstrom resolution. *Science* **1998**, *282*, 1853–1858.

(S10) Li, C.-B.; Li, Z.-J.; Yu, S.; Wang, G.-X.; Wang, F.; Meng, Q.-Y.; Chen, B.; Feng, K.; Tung, C.-H.; Wu, L.-Z. Interface-directed assembly of a simple precursor of [FeFe]-H_2ase mimics on CdSe QDs for photosynthetic hydrogen evolution in water. *Energy Environ. Sci.* **2013**, *6*, 2597–2602.

(S11) Kim, M.; Hwang, S.; Yu, J.-S. Novel ordered nanoporous graphitic C_3N_4 as a support for Pt–Ru anode catalyst in direct methanol fuel cell. *J. Mater. Chem.* **2007**, *17*, 1656–1659.

(S12) Cao, X.; Ma, J.; Lin, Y.; Yao, B.; Li, F.; Weng, W.; Lin, X. A facile microwave-assisted fabrication of fluorescent carbon nitride quantum dots and their application in the detection of mercury ions. *Spectrochim. Acta A* **2015**, *151*, 875–880.

(S13) Thomas, A.; Fischer, A.; Goettmann, F.; Antonietti, M.; Müller, J.-O.; Schlögl, R.; Carlsson, J. M. Graphitic carbon nitride materials: variation of structure and morphology and their use as metal-free catalysts. *J. Mater. Chem.* **2008**, *18*, 4893–4908.

(S14) Kasap, H.; Caputo, C. A.; Martindale, B. C.; Godin, R.; Lau, V. W.-h.; Lotsch, B. V.; Durrant, J. R.; Reisner, E. Solar-driven reduction of aqueous protons coupled to selective alcohol oxidation with a carbon nitride–molecular Ni catalyst system. *J. Am. Chem. Soc.* **2016**, *138*, 9183–9192.

(S15) Lau, V. W.-h.; Klose, D.; Kasap, H.; Podjaski, F.; Pignié, M.-C.; Reisner, E.; Jeschke, G.; Lotsch, B. V. Dark photocatalysis: storage of solar energy in carbon nitride for time-delayed hydrogen generation. *Angew. Chem. Int. Ed.* **2017**, *129*, 525–529.

(S16) Yang, W.; Godin, R.; Kasap, H.; Moss, B.; Dong, Y.; Hillman, S. A.; Steier, L.; Reisner, E.; Durrant, J. R. Electron accumulation induces efficiency bottleneck for hydrogen production in carbon nitride photocatalysts. *J. Am. Chem. Soc.* **2019**, *141*, 11219–11229.

(S17) Yamazaki, T.; Nabeshima, M.; Saito, T.; Yamaji, T.; Hayamizu, K.; Yanagisawa, M.; Yamamoto, O. Spectral Database for Organic Compounds (SDBS). *National Institute of Advanced Industrial Science and Technology (AIST), Japan, https://sdbs.db.aist.go.jp*

(S18) Jofre, F.; Anderson, M. E.; Markley, J. L. Biological Magnetic Resonance Data Bank (BMRB). *BMRB entry bmse000788, https://doi.org/10.13018/BMSE000788*

(S19) Zhao, G.; Chasteen, N. D. Oxidation of Good's buffers by hydrogen peroxide. *Anal. Biochem.* **2006**, *349*, 262–267.

(S20) Good, N. E.; Winget, G. D.; Winter, W.; Connolly, T. N.; Izawa, S.; Singh, R. M. Hydrogen ion buffers for biological research. *Biochemistry* **1966**, *5*, 467–477.

(S21) Han, Q.; Wang, B.; Gao, J.; Cheng, Z.; Zhao, Y.; Zhang, Z.; Qu, L. Atomically thin mesoporous nanomesh of graphitic C_3N_4 for high-efficiency photocatalytic hydrogen evolution. *ACS Nano* **2016**, *10*, 2745–2751.

(S22) Ran, J.; Ma, T. Y.; Gao, G.; Du, X.-W.; Qiao, S. Z. Porous P-doped graphitic carbon nitride nanosheets for synergistically enhanced visible-light photocatalytic H_2 production. *Energy Environ. Sci.* **2015**, *8*, 3708–3717.

(S23) Brown, K. A.; Dayal, S.; Ai, X.; Rumbles, G.; King, P. W. Controlled assembly of hydrogenase-CdTe nanocrystal hybrids for solar hydrogen production. *J. Am. Chem. Soc.* **2010**, *132*, 9672–9680.

(S24) Hola, K.; Pavliuk, M. V.; Nemeth, B.; Huang, P.; Zdrazil, L.; Land, H.; Berggren, G.; Tian, H. Carbon dots and [FeFe] hydrogenase biohybrid assemblies for efficient light-driven hydrogen evolution. *ACS Catal.* **2020**, *10*, 9943–9952.

(S25) Bisquert, J. Theory of the impedance of electron diffusion and recombination in a thin layer. *J. Phys. Chem. B* **2002**, *106*, 325–333.

(S26) Liu, Y.; Quiñonero, J.; Yao, L.; Da Costa, X.; Mensi, M.; Gómez, R.; Sivula, K.; Guijarro, N. Defect engineered nanostructured $LaFeO_3$ photoanodes for improved activity in solar water oxidation. *J. Mater. Chem. A* **2021**, *9*, 2888–2898.
